# TWIST1 Integrates Endothelial Responses to Flow in Vascular Dysfunction and Atherosclerosis

**DOI:** 10.1161/CIRCRESAHA.116.308870

**Published:** 2016-07-21

**Authors:** Marwa M. Mahmoud, Hyejeong Rosemary Kim, Rouyu Xing, Sarah Hsiao, Akiko Mammoto, Jing Chen, Jovana Serbanovic-Canic, Shuang Feng, Neil P. Bowden, Richard Maguire, Markus Ariaans, Sheila E. Francis, Peter D. Weinberg, Kim van der Heiden, Elizabeth A. Jones, Timothy J.A. Chico, Victoria Ridger, Paul C. Evans

**Affiliations:** From the Department of Infection, Immunity and Cardiovascular Disease, INSIGNEO Institute for In Silico Medicine, and the Bateson Centre, University of Sheffield, Sheffield, United Kingdom (M.M.M., H.R.K., S.H., J.S.-C., S.F., N.P.B., R.M., M.A., S.E.F., T.J.A.C., V.R., P.C.E.); ERASMUS MC, Rotterdam, The Netherlands (R.X., K.v.d.H.); Vascular Biology Program, Department of Surgery (A.M.) and Department of Ophthalmology (J.C.), Boston Children’s Hospital, Harvard Medical School, MA; Department of Bioengineering, Imperial College London, London, United Kingdom (P.D.W.); and Department of Cardiovascular Science, Katholieke Universiteit Leuven, Leuven, Belgium (E.A.J.).

**Keywords:** atherosclerosis, cholesterol, gastrulation, obesity, transcription factors

## Abstract

**Rationale:**

Blood flow–induced shear stress controls endothelial cell (EC) physiology during atherosclerosis via transcriptional mechanisms that are incompletely understood. The mechanosensitive transcription factor TWIST is expressed during embryogenesis, but its role in EC responses to shear stress and focal atherosclerosis is unknown.

**Objective:**

To investigate whether TWIST regulates endothelial responses to shear stress during vascular dysfunction and atherosclerosis and compare TWIST function in vascular development and disease.

**Methods and Results:**

The expression and function of TWIST1 was studied in EC in both developing vasculature and during the initiation of atherosclerosis. In zebrafish, *twist* was expressed in early embryonic vasculature where it promoted angiogenesis by inducing EC proliferation and migration. In adult porcine and murine arteries, TWIST1 was expressed preferentially at low shear stress regions as evidenced by quantitative polymerase chain reaction and en face staining. Moreover, studies of experimental murine carotid arteries and cultured EC revealed that TWIST1 was induced by low shear stress via a GATA4-dependent transcriptional mechanism. Gene silencing in cultured EC and EC-specific genetic deletion in mice demonstrated that TWIST1 promoted atherosclerosis by inducing inflammation and enhancing EC proliferation associated with vascular leakiness.

**Conclusions:**

TWIST expression promotes developmental angiogenesis by inducing EC proliferation and migration. In addition to its role in development, TWIST is expressed preferentially at low shear stress regions of adult arteries where it promotes atherosclerosis by inducing EC proliferation and inflammation. Thus, pleiotropic functions of TWIST control vascular disease and development.

## Abstract 2

Supplemental Digital Content is available in the text.

Endothelial cells (EC) are exquisitely sensitive to shear stress (mechanical drag), which is imposed on the vessel wall by flowing blood. Although atherosclerosis is promoted by systemic risk factors (eg, cholesterol, smoking, obesity, and age), it develops preferentially near branches and bends exposed to complex blood flow that generates shear stress with low-magnitude and significant variation in direction (eg, oscillations and tangential shear). By contrast, atheroprotected sites are exposed to shear stress with high magnitude and uniform direction.^1–4^ Shear stress controls fundamental processes in EC, including inflammation, proliferation, and migration. Low shear stress promotes atherosclerosis by priming EC for enhanced expression of inflammatory molecules (eg, intercellular adhesion molecule-1 [ICAM-1] and vascular cell adhesion molecule-1 [VCAM-1]) that coordinate the migration of leukocytes from the blood stream to the vascular wall.^3–8^ Low shear stress is also associated with enhanced EC proliferation,^9–11^ a process where EC lose contact with neighboring cells, thereby enhancing vascular permeability to cholesterol-rich lipoproteins to drive lesion formation.^12^ Shear stress controls EC physiology, in part, via transcriptional and post-transcriptional mechanisms that are incompletely characterized.^4–6,13–18^

A recent microarray study from our group demonstrated that the helix-loop-helix transcription factor TWIST1 and the zinc finger transcription factor GATA4 were enriched in EC at low shear stress atheroprone regions of the aorta (J. Serbanovic-Canic and P.C. Evans, unpublished data, 2016). *twist* was originally identified in *Drosophila* embryos where it controls gastrulation and other fundamental developmental processes.^19^ In vertebrates, TWIST regulates multiple diverse activities including development,^20,21^ epithelial–mesenchymal transition,^22^ and tumor metastasis.^23^ GATA4 is also a key regulator of development. It controls cardiac specification^24^ and genetic deletion of GATA4 in mice led to multiple cardiac phenotypes.^25^ Of note, TWIST and GATA4 exert overlapping functions during atrioventricular valve development by inducing endothelial–mesenchymal transition (EndoMT), which describes a program of phenotypic changes including enhanced EC proliferation and migration.^21,26^ Although GATA4 and TWIST have well-recognized roles in some aspects of embryogenesis, their potential function in vascular development and disease is unknown.

Here, we demonstrate for the first time that low shear stress promotes GATA4-dependent induction of TWIST1 in EC. Studies using zebrafish revealed that *twist* was expressed in early embryonic vasculature where it promoted angiogenic sprouting by inducing EC proliferation and migration. In adult mammalian arteries, TWIST1 was expressed preferentially at atheroprone sites exposed to low shear stress where it promoted the development of atherosclerosis by inducing inflammation and EC proliferation. We conclude that TWIST is a shear stress–regulated transcription factor that regulates angiogenesis in embryos and drives focal EC dysfunction and atherosclerosis in adult arteries.

## Methods

### Mice

Male mice between 2 and 3 months of age were used. For cell tracking studies, transgenic Rosa26-tdTomato mice^27^ were crossed with endothelial-SCL-Cre-ER^T^ mice containing a tamoxifen-inducible EC-specific Cre.^28^ To activate Cre, tamoxifen was administered for 5 consecutive days (2 mg/mouse/d). Mice with conditional deletion of TWIST1 (*TWIST1*^*cKO*^) were generated by crossing Tie2-Cre–expressing mice (Jackson Laboratory stock 004128) with TWIST1 floxed mice (*TWIST1*^*flox*|*flox*^).^29^ Mice with conditional EC deletion of GATA4 (called GATA4^*cKO*^) were generated by crossing endothelial-SCL-Cre-ER^T^ mice with GATA4 floxed mice *(GATA4*^*flox|flox*^*).* Constrictive cuffs were applied to the right carotid artery of isoflurane-anesthetized mice following published methods.^6,30^ The expression levels of specific proteins were assessed in EC by en face staining as previously described.^5,6^ Hypercholesterolemia was induced by tail vein injection of adeno-associated virus containing a gain-of-function mutated version of proprotein convertase subtilisin/kexin type 9 (rAAV8-D377Y-mPCSK9) gene followed by exposure to a western diet for 6 weeks as previously described.^31^ Lesions were detected by Oil Red O staining.

### Cultured EC

Human umbilical vein EC and porcine aortic EC were isolated and cultured.^32^ Gene silencing was performed using 2 different small interfering RNAs (siRNAs) against TWIST1 ((Silencer Select S14523, Ambion, and L-006434-00-0005 ON-TARGETplus SMARTpool; Dharmacon) or GATA4 (Silencer Select s5603, Ambion, and L-008244-00-0005 ON-TARGETplus SMARTpool; Dharmacon). They were exposed to flow using an orbital shaking system or Ibidi parallel-plate system.^32,33^ Quantitative RT-PCR, immunofluorescent staining and chromatin immunoprecipitation,^6^ and assays of permeability^34^ were performed as described.

### Zebrafish Embryos

Studies were performed using wild-type or transgenic zebrafish lines, *Tg(fli1:EGFP*) (endothelial EGFP), *Tg(kdrl:NLS-EGFP*) (endothelial nuclear EGFP), or *Tg(gata1:dsRed*) (red blood cell expression of dsRed). Whole-mount in situ hybridization was performed as described.^35^ The *twist1b* gene was mutated using the CRISPR/Cas9 system and *twist1a* expression was reduced using a translation-blocking morpholino.^36^

### Statistics

Differences between samples were analyzed using an unpaired or paired Student *t* test or ANOVA (*P<0.05, **P<0.01, and ***P<0.001).

## Results

### Low Shear Stress Induced TWIST1 via a GATA4-Dependent Mechanism

**Figure 1.**
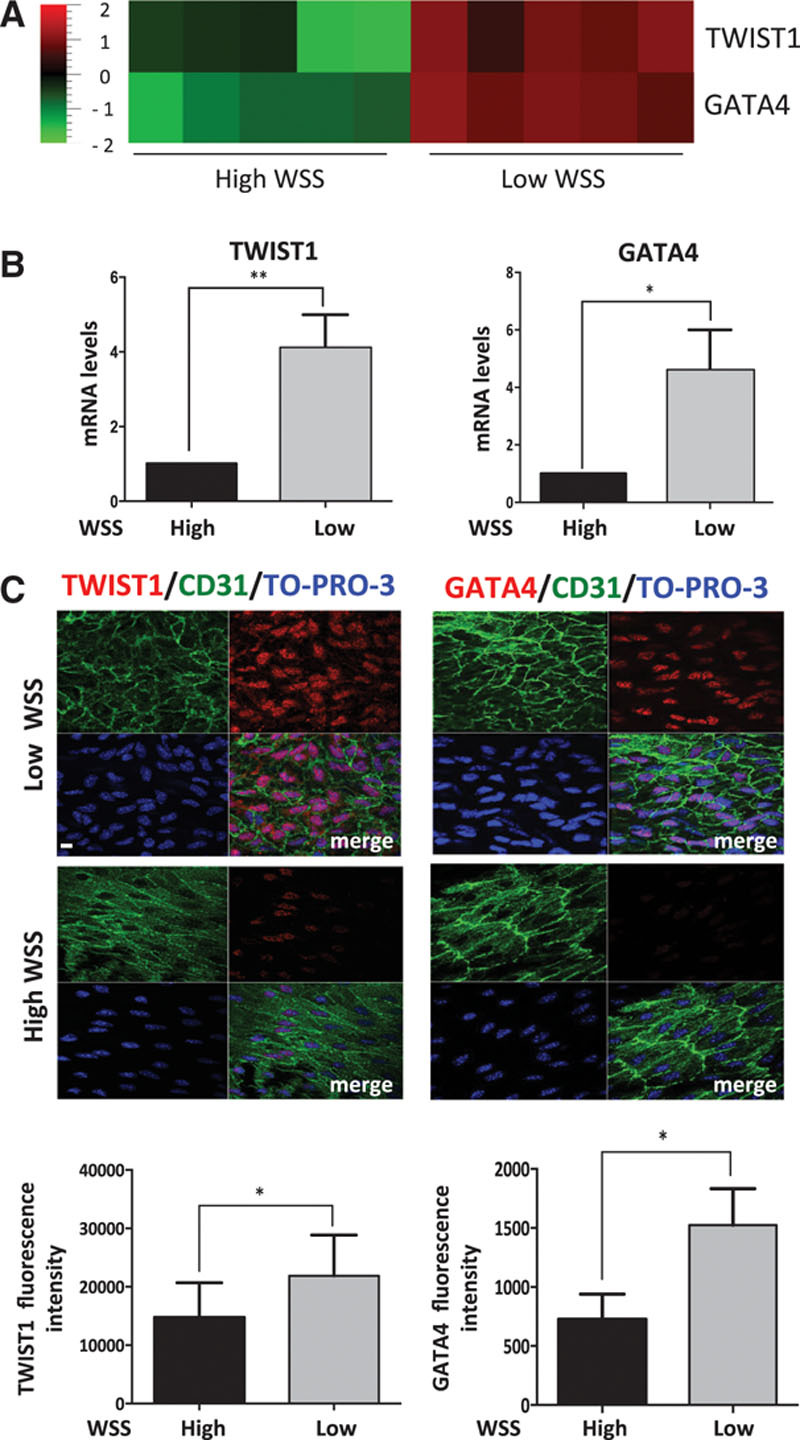
**TWIST1 and GATA4 were preferentially expressed at low shear atherosusceptible sites**. **A** and **B**, Expression of TWIST1 and GATA4 was studied at low wall shear stress (WSS; inner curvature) and high WSS (outer curvature) regions of the porcine aorta. **A**, Differentially expressed genes were identified using microarrays (n=5 pigs) and the expression patterns of TWIST1 and GATA4 are presented as a heat map with red indicating enrichment in expression. **B**, Results were validated by qRT-PCR of a different cohort of pigs (n=5). The expression level at the low WSS site is presented relative to the expression at the high WSS site (normalized to 1; dotted line). Mean levels±SEM are shown. **C**, Expression levels of TWIST1 or GATA4 in EC were assessed by en face staining of low (inner curvature) or high (outer curvature) WSS regions of the aorta in C57BL/6 mice (red). Endothelial cells (ECs) were identified by costaining with anti-CD31 antibodies conjugated to FITC (fluorescein isothiocyanate; green). Cell nuclei were identified using TO-PRO-3 (blue). Representative images and quantification of TWIST1 or GATA4 (mean±SEM) are shown. *P<0.05 and **P<0.01, using a paired *t* test.

A microarray study from our laboratory comparing EC at low shear stress (inner curvature of aortic arch) and high shear stress (outer curvature) regions in the porcine aorta revealed >800 differentially expressed genes (J. Serbanovic-Canic and P.C. Evans, unpublished data, 2016). They included TWIST1 and GATA4 that were enriched at the low shear stress site (subanalysis shown in Figure 1A). This observation was validated by quantitative polymerase chain reaction (qPCR) studies of an independent cohort of pigs (Figure 1B). Similarly, en face staining of the murine aortic endothelium demonstrated that TWIST1 and GATA4 proteins were expressed at higher levels at the inner curvature of the aortic arch (low shear stress) compared with the outer curvature (high shear stress; Figure 1C). Moreover, both of these transcription factors localized to the nucleus, suggesting that they are active at the low shear site.

**Figure 2.**
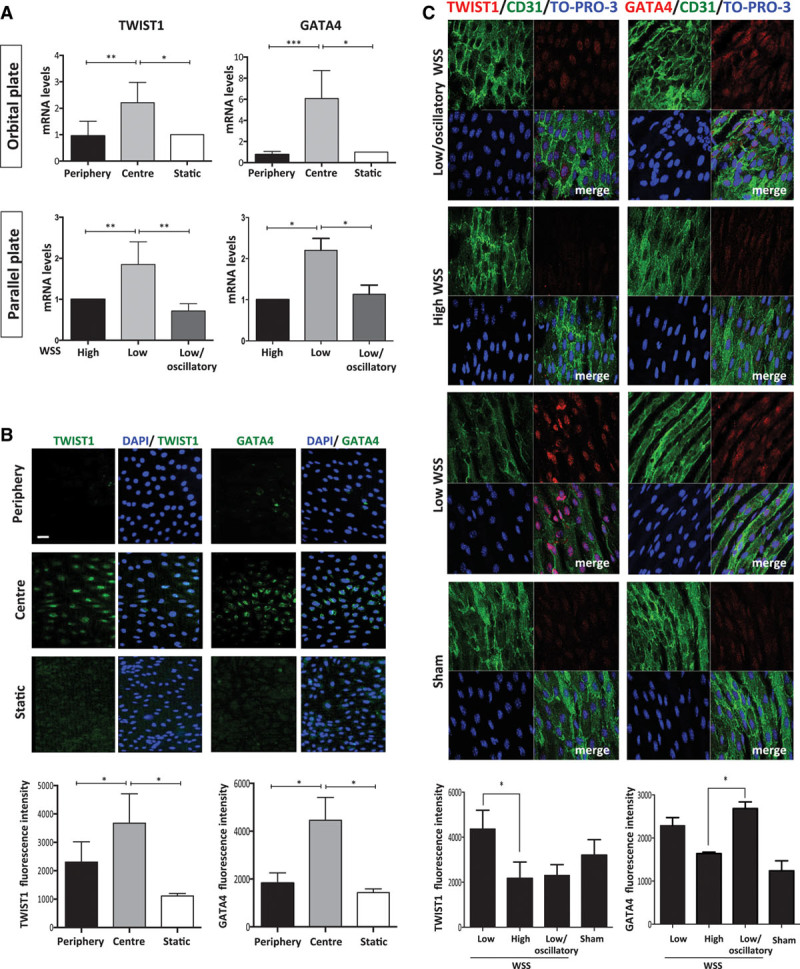
**Low shear stress induced TWIST1 and GATA4. A**, Human umbilical vein endothelial cells (HUVECs) were exposed to orbital flow to generate low (center) or high (periphery) wall shear stress (WSS) or were cultured under static conditions. Alternatively, HUVECs were exposed to high, low, or low/oscillatory WSS using a parallel-plate system. After 72 h, levels of TWIST1 or GATA4 transcripts were quantified by qRT-PCR. **B**, HUVECs were exposed to orbital flow to generate low (center) or high (periphery) WSS for 72 h or were cultured under static conditions. Expression of TWIST1 and GATA4 was determined by immunofluorescent staining (green) and costaining using DAPI (4′,6-diamidino-2-phenylindole; blue). Bar=50 μm. Fluorescence intensities were measured in multiple cells in 3 independent experiments, and mean values±SEM are shown. **C**, Flow-altering, constrictive cuffs were placed on the right carotid arteries of C57BL/6 mice. They generated anatomically distinct regions exposed to low, high, and low oscillatory WSS (as indicated). Right (experimental) and left (sham-operated) carotid arteries were harvested after 14 days, and en face staining was performed using anti-TWIST1 or anti-GATA4 antibodies (red), anti-CD31 antibodies conjugated to FITC (fluorescein isothiocyanate; green), and the nuclear counter stain TO-PRO-3 (blue). Representative images and quantification of TWIST1 or GATA4 expression (mean±SEM) are shown. Bar=10 μm. Data were pooled from 5–6 independent experiments. *P<0.05 and **P<0.01, using a 1-way ANOVA.

Atherosusceptible regions of arteries are associated with increased inflammation and altered transport of substances to and from the arterial wall as well as low shear stress. Therefore, we used in vitro and in vivo models to examine whether shear stress per se is responsible for enhanced expression of TWIST1 and GATA4. Cultured EC were exposed to flow using 2 complementary systems, an orbital-plate and a parallel-plate apparatus. Computational fluid dynamic analysis demonstrated that the orbiting 6-well plate system generates low mean shear stress (4.8 dyn/cm^2^) with rapid variations in direction at the center and high mean shear stress (11.1 dyn/cm^2^) with uniform direction at the periphery.^32,33^ Using the orbital system, TWIST1 and GATA4 expression was elevated in Human umbilical vein EC or porcine aortic EC exposed to low (center) compared with high shear stress (periphery) or static conditions (Figure 2A, top, and Figure 2B; Online Figure I). Indeed, the majority of cells exposed to low shear stress expressed TWIST1 and GATA4, which localized almost exclusively to the nucleus (Figure 2B). To delineate between the effects of shear stress magnitude and direction, a parallel-plate system was used to compare gene expression under low oscillatory (±4 dyn/cm^2^), low unidirectional (4 dyn/cm^2^), and high unidirectional (13 dyn/cm^2^) flow. It revealed that TWIST1 and GATA4 expression was higher in EC exposed to low compared with high or low oscillatory shear stress (Figure 2A, bottom), indicating that low-magnitude shear stress drives GATA4 and TWIST1 expression in cultured EC. We determined whether shear stress regulates GATA4 and TWIST1 expression in vivo by modifying flow in the murine carotid artery. This was achieved using a constrictive cuff that causes tapering of the lumen to generate high shear stress at the stenosis, low shear stress upstream, and low/oscillatory shear stress downstream.^6,30^ Cuff placement for 14 days led to enhanced expression of GATA4 and TWIST1 at the low shear stress site (Figure 2C), which is consistent with our in vitro data. By contrast, GATA4 was induced in the absence of TWIST1 at the low oscillatory shear stress site (Figure 2C). This apparent discrepancy with the in vitro data (where low oscillatory shear had no effect on GATA4 expression) may be because of differences in the frequency of oscillation or features of the flow waveform between in vitro and in vivo systems.^30^

**Figure 3.**
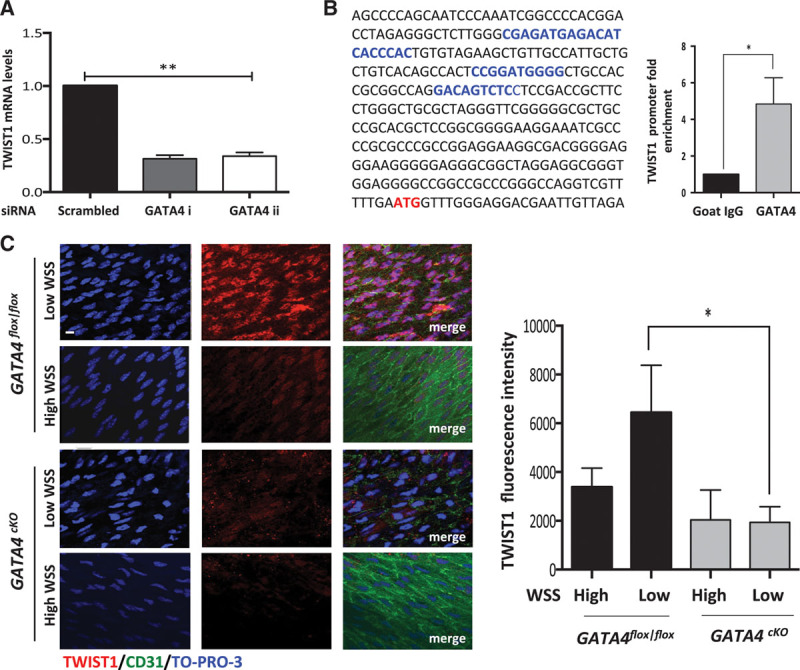
**GATA4 induced TWIST1 in response to low shear stress. A**, Human umbilical vein endothelial cells (HUVECs) were transfected with 2 different small interfering RNAs (siRNAs) targeting GATA4 or with scrambled sequences and exposed to orbital flow for 72 h. Cells exposed to low wall shear stress (WSS; center of well) were collected, and TWIST1 transcript levels were quantified by qRT-PCR. **B**, GATA4-binding sites (A/T)GATAA(G) were predicted in the TWIST1 5′ untranslated region, and their position (blue text) in relation to the translational start site (red text) is shown. HUVECs were exposed to orbital flow for 72 h before chromatin immunoprecipitation using anti-GATA4 or irrelevant isotype control antibodies. The levels of TWIST1 promoter DNA were assessed by qPCR and fold enrichment in anti-GATA4 compared with control precipitates was calculated. Mean levels±SEM are shown. **A** and **B**, Data from at least 3 independent experiments were pooled. *P<0.05 and **P<0.01 using an unpaired *t* test. **C**, Endothelial cells (ECs) at low WSS (susceptible) or high WSS (protected) regions of the aorta in GATA4^cKO^ or GATA4^fl/fl^ mice were studied by en face staining (n=5). TWIST1 (red) expression was assessed by en face staining. Representative images are shown, and TWIST1 expression was quantified (mean fluorescence±SEM is shown). Bar=10 μm. *P<0.05 using a 2-way ANOVA.

We hypothesized that GATA4 and TWIST1 may be cross-regulated because both were induced by low wall shear stress (WSS; Figures 1 and 2) and have overlapping functions in other systems.^26^ Potential cross-regulation was tested by silencing of GATA4 or TWIST1 using 2 different siRNA sequences and validation by qPCR and Western blotting (Online Figure II). GATA4 was required for TWIST1 expression in EC exposed to low shear stress (Figure 3A), whereas TWIST1 silencing did not alter GATA4 expression (data not shown). We hypothesized that GATA4 may positively regulate transcription of TWIST1 because putative GATA4-binding sites were identified in its promoter (Figure 3B). Consistent with this, chromatin immunoprecipitation followed by qPCR demonstrated that TWIST1 promoter sequences coprecipitate with anti-GATA4 antibodies but not with isotype-matched irrelevant IgG (Figure 3B), indicating that GATA4 binds to the promoter region of TWIST1 to induce transcriptional activation. To validate these observations, we assessed the effects of genetic deletion of GATA4 on endothelial expression of TWIST1 in the murine aorta. GATA4 deletion from EC was achieved by crossing GATA4^flox/flox^ mice with endothelial-SCL-Cre-ER transgenics followed by 5 days of tamoxifen treatment (generating GATA4^cKO^). To validate genetic deletion, it was shown by en face staining that GATA4 expression was absent from EC in GATA4^cKO^ mice (Online Figure IIIB). En face staining revealed that the expression of TWIST1 at the low shear stress site was reduced in GATA4^cKO^ compared with GATA4^flox/flox^ mice (Figure 3C), indicating that GATA4 positively regulates TWIST1 in atherosusceptible endothelium. Thus, we conclude that GATA4 induces TWIST1 in cells exposed to low shear stress, and this pathway is suppressed in cells exposed to high shear stress. Our observation that TWIST1 was not expressed in GATA4-expressing EC that were exposed to low oscillatory shear stress in vivo (Figure 2C) indicates that factors other than GATA4 are required for TWIST1 expression. Candidate coregulators include Notch1 that positively regulates TWIST1 in other systems^37,38^ and was induced by low (but not low oscillatory shear stress) in modified carotid arteries (Online Figure IV). Collectively, our in vitro and in vivo data reveal that low-magnitude WSS positively regulates TWIST1 expression via a transcriptional pathway that requires GATA4 among other factors. We conclude that low shear induces TWIST1 via increasing its synthesis but cannot rule out additional mechanisms that reduce the rate of degradation.

Finally, we used a Cre-based cell-tracking system to investigate whether low shear stress induces TWIST1 expression in fully differentiated EC and/or whether it promotes the homing of TWIST1-positive endothelial progenitor cells to the vascular wall. Cell tracking was performed using transgenic SCL-Cre-ER^T^/R26R-tdTomato mice where EC were labeled with tdTomato in response to tamoxifen treatment. Notably, transient tamoxifen treatment allowed mature EC that reside in vessels (tdTomato-positive; Online Figure V) to be distinguished from those generated by subsequent homing of progenitor cells (tdTomato-negative).^28^ Using this system, we labeled EC with tdTomato before placement of a constrictive cuff on the carotid artery for 2 weeks and analysis of GATA4 and TWIST1 expression. We observed that EC at the low shear stress site that expressed GATA4 or TWIST1 were tdTomato-positive (Online Figure VB), indicating that GATA4–TWIST1 signaling can be induced by low shear stress in fully differentiated EC of adult arteries and that progenitor EC homing was negligible.

### Twist Promoted EC Proliferation and Migration in Embryonic Vasculature

**Figure 4.**
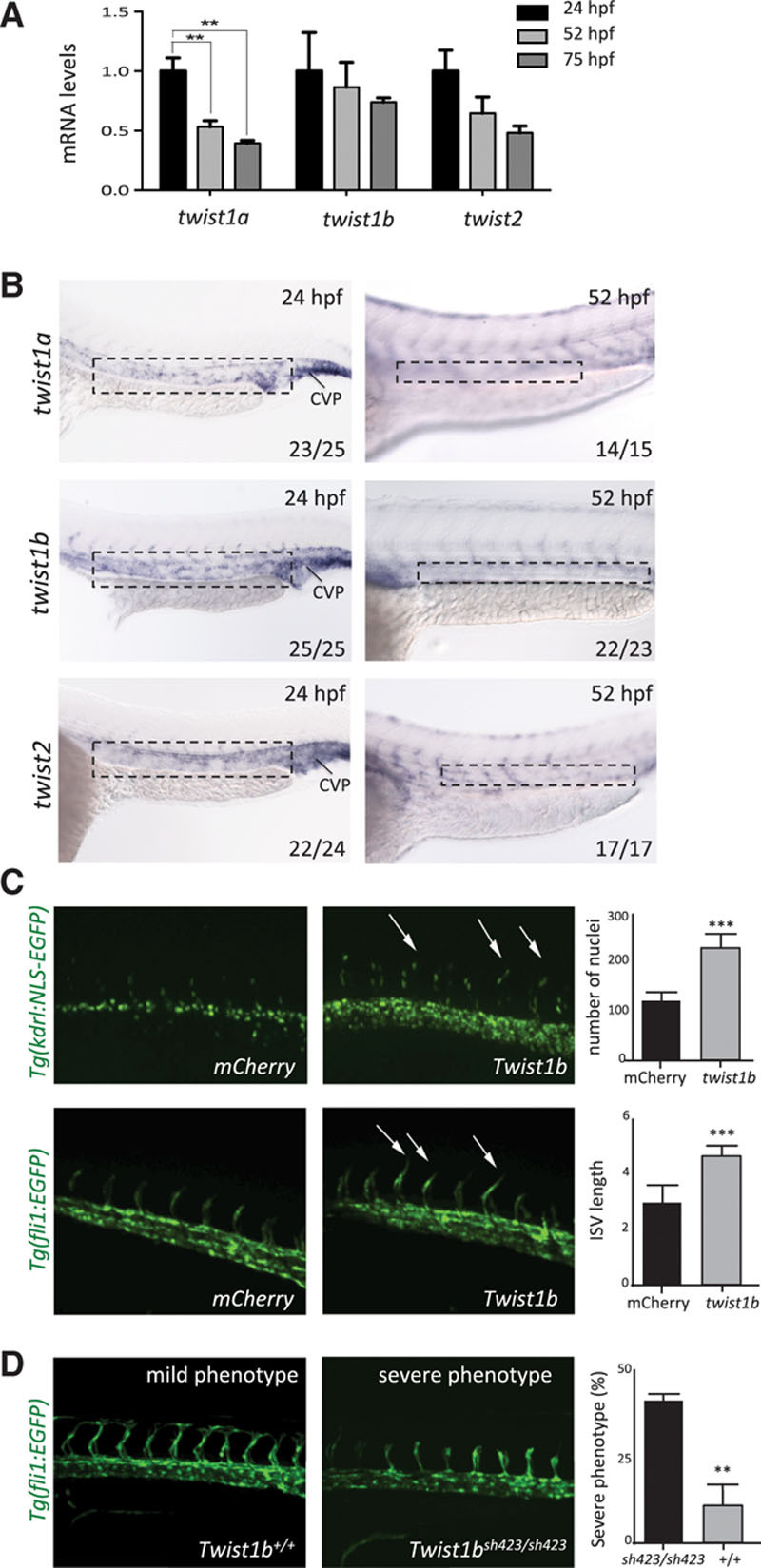
***twist* promoted intersegmental vessel sprouting in zebrafish embryos. A**, The expression of *twist1a*, *twist1b*, and *twist2* was studied at 24 to 75 hours post fertilization (hpf) by qPCR of the trunk and tail of embryos. Data were pooled from n≥15 embryos studied in 3 independent experiments and mean values±SEs are shown. **B**, The expression of *twist1a*, *twist1b*, and *twist2* was studied at 24 and 52 hpf by in situ hybridization. Data are representative of the majority of embryos analyzed (proportion indicated lower right of each part) and were closely similar in at least 3 independent experiments. Higher magnification insets are shown (marked in **top**). Bar=100 µm. **C**, Zebrafish embryos (wild-type, *Tg*(*fli1:EGFP*), or *Tg*(*kdrl:NLS-EGFP*)) were treated with *twist1b* mRNA (to enforce expression) or treated with mCherry mRNA as a control. They were studied at 24 to 27 hpf using confocal microscopy to visualize endothelial cell (EC) nuclei (*Tg*(*kdrl:NLS-EGFP*); **top**) or angiogenic sprouts (*Tg(fli1:EGFP*); **bottom**; arrows). Representative images are shown. Cell numbers and the length of intersegmental vessels (ISVs; third to fifth vessels in the field view) were quantified in multiple embryos, and mean values±SEM are shown (**right**). **D**, The *twist1b* coding sequence was mutated by introduction of a 4 bp deletion causing a frameshift and premature stop (mutant allele designated *twist1b*^*sh423*^). *twist1b*^*sh423/+*^ *Tg*(*fli1:EGFP*) fish were incrossed, and embryos were treated with a morpholino directed against *twist1a*. Sprouting of ISV was assessed at 34 hpf. Embryos were classified into those that displayed minimal sprouting (severe phenotype) and those with intermediate levels of sprouting (mild phenotype). Genotyping was subsequently performed, and the proportion of *twist1b* homozygous mutants (*twist1b*^*sh423/sh423*^) and *twist1b* homozygous wild-types (*twist1b*^*+/+*^) in the severe phenotype group was calculated (% indicated). Representative images are shown. Data were closely similar in 3 independent experiments. ***P<0.001 and **P<0.01 using a 1-way ANOVA (**A**) or unpaired *t* test (**C** and **D**). CVP indicates caudal vein plexus.

Given that TWIST is expressed in embryogenesis^20,21^ and at atheroprone regions of adult arteries (Figure 1), we wished to compare its function in EC during vascular development and disease. The potential role of TWIST in vascular development was studied using zebrafish that possess 3 othologs of mammalian TWIST genes: *twist1b*, *twist1a*, and *twist2.* Quantitative RT-PCR and in situ hybridization revealed that each of them was expressed in the trunk vasculature at 24 hours post fertilization and subsequently declined in expression at later developmental stages (Figures 4A and 4B). The function of *twist* was studied by enhancing its expression in vessels via injection of mRNA (gain of function). Transgenic fish expressing green fluorescent protein in EC (*Tg*(*fli1:EGFP*) or endothelial nuclei (*Tg*(*kdrl:NLS-EGFP*) were used to facilitate visualization of the vasculature and cell counting, respectively. Dynamic imaging and analysis of fixed embryos demonstrated that *twist1b* overexpression led to enhanced sprouting of intersegmental vessels associated with increased EC migration and proliferation (Online Movies I and II; Figure 4C). Conversely, suppression of *twist1* expression (loss-of-function) reduced intersegmental vessel formation (Figure 4D). The latter study was performed by mutating *twist1b* using CRISPR/Cas9 genome editing to introduce a 4 bp deletion that caused a frame shift and introduced a stop codon before the helix-loop-helix domain (designated *twist1b*^*sh423*^). Intersegmental vessel sprouting was not altered in *twist1b*^*sh423*^ mutants (data not shown), and we hypothesized that this was because of compensation from *twist1a* that is closely homologous. To test this, *twist1b*^sh423/+^ fish were incrossed to generate *twist1b*^sh423/*sh423*^ mutant (25%), heterozygotes (50%), and *twist1b*^+/+^ wild-type (25%) embryos that were subsequently treated with a morpholino directed against *twist1a*. Embryos displayed variable phenotypes and were classified into those with minimal sprouting (severe phenotype) and those with intermediate levels of sprouting (mild phenotype). Notably, the severe phenotype was significantly enriched in the homozygous mutant group compared with wild types, indicating that mutation of *twist1b* enhanced the *twist1a* knock down effect (Figure 4D). Overall, these data suggest that *twist1b* and *twist1a* overlap at a functional level to promote angiogenic sprouting. Similar observations were made in a different vascular bed, the developing subintestinal vein. At this site, *twist* genes were expressed in early developing vasculature and enforced expression of *twist1b* enhanced angiogenic sprouting (Online Figure VI). Collectively, these data indicate that *twist* promotes developmental angiogenesis by inducing EC migration and proliferation.

### TWIST1 and GATA4 Promote EC Proliferation and Inflammation Under Low Shear Stress Conditions

**Figure 5.**
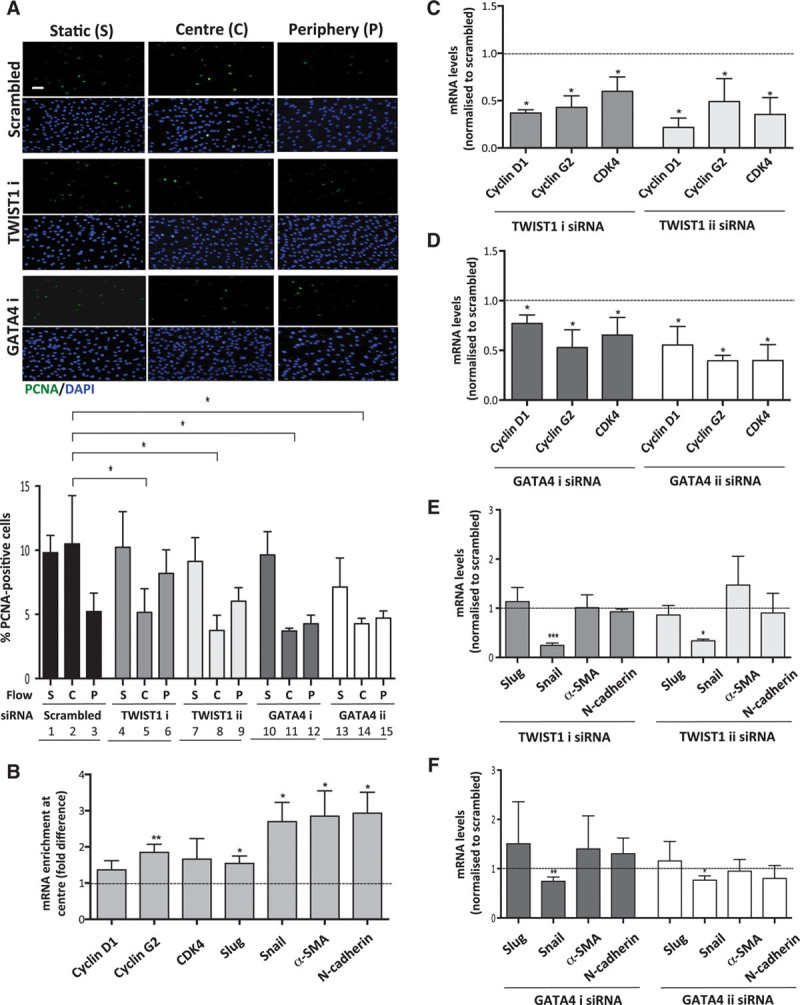
**TWIST1 and GATA4 promoted proliferation in endothelial cells (ECs) exposed to low shear stress**. Human umbilical vein endothelial cells (HUVECs) were treated with 2 different small interfering RNAs (siRNAs) targeting TWIST1 or GATA4 (designated i and ii) or with scrambled nontargeting siRNA or remained untransfected. Cells were subsequently cultured in 6-well plates before exposure to orbital flow to generate low (center, C) or high (periphery, P) wall shear stress (WSS) for 72 h. Alternatively, cells were maintained under static (S) conditions. **A**, Cell proliferation was quantified by immunofluorescent staining using anti-proliferating cell nuclear antigen (PCNA) antibodies and costaining using DAPI (4′,6-diamidino-2-phenylindole). Images are representative of those generated in 3 independent experiments using 1 version of the gene-specific siRNA or scrambled control sequences (bar=50 μm). The percentage of PCNA-positive cells were calculated for multiple fields of view in at least 3 independent experiments, and mean values±SEM are shown. *P<0.05 using a 2-way ANOVA. **B–F**, The expression of cell cycle regulators and endothelial–mesenchymal transition genes was quantified using qRT-PCR. **B**, The expression level in cells at the center (low WSS) is presented relative to the expression at the periphery (high WSS; normalized to 1; dotted line). **C–F**, Transfected cells were exposed to low WSS (center). The expression level in cells transfected with gene-targeting siRNA is presented relative to the expression in cells transfected with scrambled control siRNA (normalized to 1; dotted line). Data were pooled from 3 independent experiments, and mean values±SEM are shown. *P<0.05, **P<0.01, and ***P<0.001 using an unpaired *t* test.

We wished to know whether GATA4–TWIST1 signaling influences EC dysfunction and the initiation of atherosclerosis at low shear sites. This was initially studied using cultured EC exposed to flow under well-controlled experimental conditions. We hypothesized that TWIST and GATA4 promote proliferation in EC exposed to low shear because they regulate cell cycle progression in other contexts.^20,21^ Consistent with this concept, gene silencing of TWIST1 or GATA4 using 2 different siRNAs significantly reduced proliferation of EC exposed to low shear using the orbital system (Figure 5A, center; compare 2 with 5, 8, 11, and 14) but did not alter EC exposed to high shear (periphery; compare 3 with 6, 9, 12, and 15) or static conditions (compare 1 with 4, 7, 10, and 13). These data were validated using a parallel-plate system to demonstrate that silencing of TWIST1 or GATA4 reduced proliferation of EC that were exposed to low but not high shear stress conditions (Online Figure VII). Thus, we conclude that low shear stress activation of GATA4–TWIST1 signaling drives EC proliferation.

We investigated whether the mechanism for enhanced proliferation involved induction of regulators of cell division. qPCR revealed that cyclin D1, cyclin G2, and cyclin-dependent kinase 4 were induced in cultured EC by the application of low shear stress (Figure 5B), and their expression was reduced by silencing of GATA4 or TWIST1 using 2 different siRNAs to ensure specificity (Figures 5C and 5D). Thus, GATA4–Twist1 signaling under low shear stress induces cyclins and cyclin-dependent kinase 4. We also investigated whether GATA4 and TWIST1 promote the expression of regulators of EndoMT (a proproliferative cellular transition) under low shear stress conditions. qPCR demonstrated that the expression of Slug, Snail, N-cadherin, and α-smooth muscle actin was elevated in EC exposed to low WSS compared with EC exposed to high WSS (Figure 5B). Silencing of GATA4 or TWIST1 (using 2 different siRNAs) significantly reduced the expression of Snail but did not influence Slug, N-cadherin, or α-smooth muscle actin (Figures 5E and 5F). Thus, we conclude that GATA4 and TWIST1 are required for the induction of Snail but that other pathways are also necessary for EndoMT under low shear stress. Collectively, our observations reveal that GATA4–TWIST1 signaling promotes EC proliferation under low shear conditions by inducing cyclins and regulators of EndoMT.

Atherosclerosis is driven by vascular inflammation involving EC expression of adhesion molecules and cytokines. The influence of GATA4 and TWIST1 on inflammatory activation was studied using cultured EC exposed to low or high shear stress. qPCR revealed that inflammatory VCAM-1 and ICAM-1 were expressed at higher levels in EC exposed to low shear (center of orbiting plate) compared with high shear, and their expression under low shear was reduced by silencing of GATA4 or TWIST1 (Online Figure VIII). Thus, GATA4–TWIST11 signaling under low shear stress induces inflammatory adhesion molecules. We also assessed whether GATA4–TWIST1 signaling influences the expression of several molecules involved in mechanosensing (Piezo, CD31, and VEGFR2). However, the expression of Piezo, CD31, and VEGFR2 was not significantly altered under low or high shear stress (Online Figure IX) or by silencing of GATA4 or TWIST1 (Online Figure IX), suggesting that GATA4–TWIST1 signaling may not alter the responsiveness of EC to shear stress.

**Figure 6.**
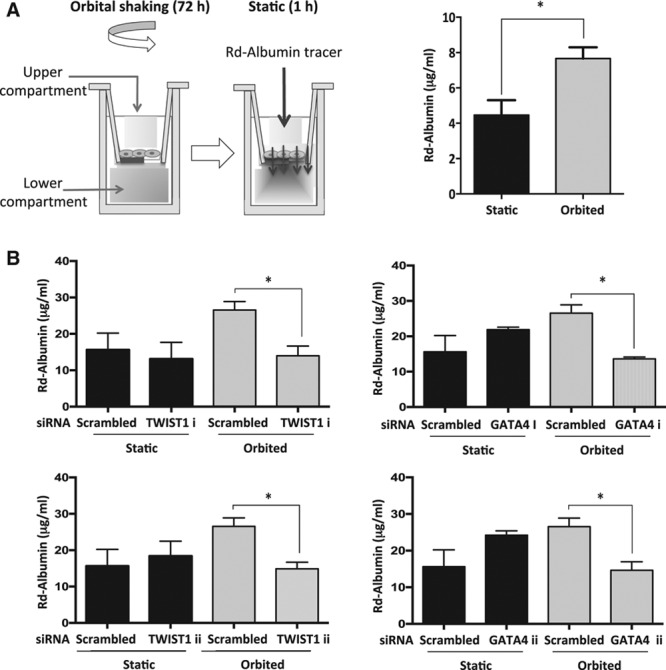
**TWIST1 and GATA4 promoted permeability in endothelial cells (ECs) exposed to low shear stress.** The influence of TWIST1 and GATA4 on EC permeability under low wall shear stress (WSS) was studied. **A**, Cells cultured on transwell inserts were exposed to orbital flow (low WSS) or static conditions for 72 h before assessment of endothelial permeability under static conditions using rhodamine (Rd) albumin as a tracer. A schematic is shown (**left**). The concentration of Rd-albumin in the lower compartment was measured, and mean values±SEM are shown (**right**). **B**, Human umbilical vein endothelial cells (HUVECs) were treated with 2 different small interfering RNAs (siRNAs) targeting TWIST1 or GATA4 (designated i and ii) or with scrambled nontargeting siRNA. Transfected cells cultured on transwell inserts were exposed to orbital flow (low WSS) or static conditions for 72 h before assessment of endothelial permeability using Rd-albumin. The concentration of Rd-albumin in the lower compartment was measured in 3 independent experiments, and mean values±SEM are shown. *P<0.05 using a paired *t* test.

Given the links between EC turnover, inflammatory activation, and vascular leakiness,^12^ we next determined whether GATA4 and TWIST1 regulate EC permeability using EC cultured on transwell inserts. Exposure of EC to low shear stress (orbited) for 72 hours enhanced permeability to rhodamine albumin compared with static conditions as previously described^34^ (Figure 6A). Silencing of TWIST1 or GATA4 (using 2 different siRNAs) reduced permeability in EC exposed to low WSS (orbited) but not in cells exposed to static conditions (Figure 6B), indicating that both genes promote permeability in response to low shear stress. Thus, TWIST1 and GATA4 are positive regulators of proliferation, inflammatory activation, and permeability under low shear conditions.

### TWIST1 and GATA4 Promote Atherogenesis by Enhancing EC Proliferation and Inflammation at Low Shear Stress Regions

**Figure 7.**
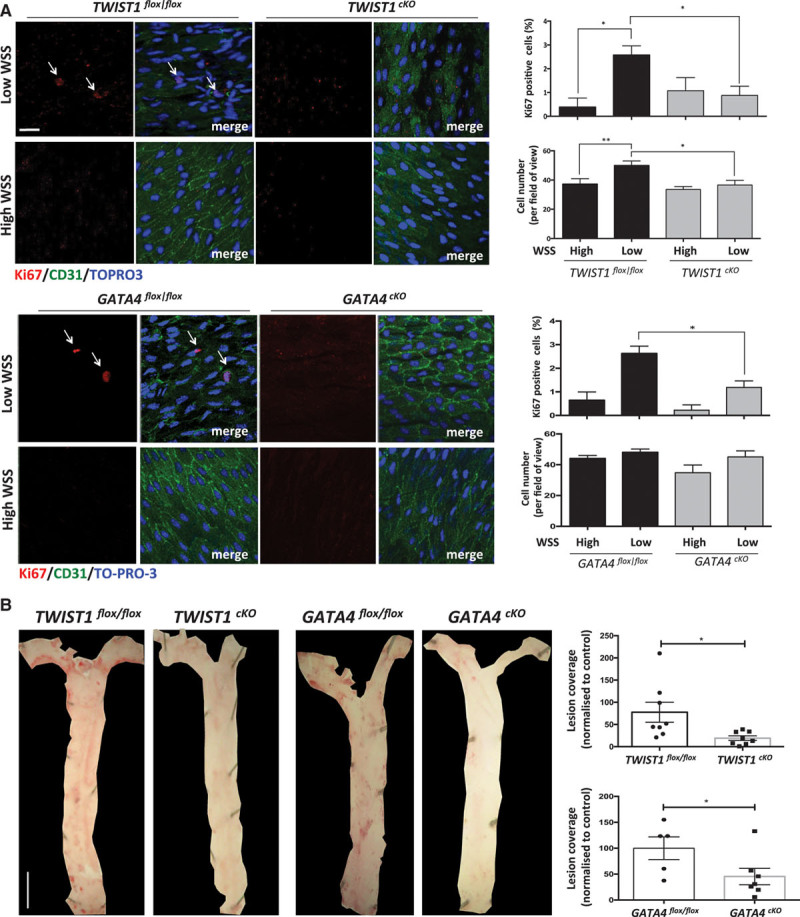
**TWIST1 and GATA4 promote endothelial cell (EC) proliferation and lesions at low shear stress sites. A**, EC at low wall shear stress (WSS; susceptible) or high WSS (protected) regions of the aorta were studied by en face staining in TWIST1^cKO^ or TWIST1^flox/flox^ mice (**top**) or in GATA4^cKO^ or GATA4^flox/flox^ mice (**bottom**). EC proliferation was quantified by anti-Ki67 staining (red). EC were identified by costaining with anti-CD31 antibodies (green). Cell nuclei were identified using TOPRO3 (blue). The proportion of Ki67-positive cells, and number of cells was calculated and mean values±SEM are shown. Bar=10 μm. Data were pooled from 5 independent experiments. **B**, TWIST1^cKO^ or TWIST1^flox/flox^ mice (**right**) or GATA4^cKO^ or GATA4^flox/flox^ mice (**left**) were treated with AAV-PCSK9. After 1 wk, they were exposed to a Western diet for 6 wk. Lesions were stained using Oil Red O and quantified using image J software. Representative images are shown (Bar=1 mm). The percentage of lesion coverage was calculated. Data were pooled from multiple mice and mean values±SEM are shown. **P<0.01 and *P<0.05 using a 2-way ANOVA (**A**) or unpaired *t* test (**B**).

Given their roles in regulating inflammation and EC proliferation in response to low shear stress, we hypothesized that GATA4 and TWIST1 may influence the initiation of atherosclerosis. Thus, the function of both molecules was studied at low shear stress sites in adult murine arteries using genetic deletion approaches. TWIST1 was deleted from EC by crossing floxed mice (TWIST1^flox/flox^) with Tie2-Cre transgenics (generating TWIST1^cKO^; Online Figure IIIA), whereas GATA4 deletion from EC was studied using GATA4^cKO^ mice (as above, Online Figure III). En face staining of Ki67 revealed that EC proliferation was enhanced in EC exposed to low compared with high shear stress as reported,^11^ and deletion of GATA4 or TWIST1 from EC significantly reduced proliferation at low shear stress sites in the aorta (Figure 7A). Deletion of TWIST1 from EC reduced the number of cells per field of view at the low shear site, whereas deletion of GATA4 did not (Figure 7A), suggesting that these molecules may have divergent effects on factors that influence cell density, for example, viability, migration, or shape. Parallel en face staining studies demonstrated that inflammatory ICAM-1 expression was enhanced at the low shear site, and its expression was reduced by deletion of TWIST1 from EC (Online Figure VIII). Plaque formation was studied by inducing hypercholesterolemia via adeno-associated virus delivery of PCSK9 followed by high fat feeding for 6 weeks. Genetic deletion of GATA4 or TWIST1 in EC reduced lesion area in the aorta, indicating that GATA4–TWIST1 signaling contributes to atherosclerosis (Figure 7B). It is unlikely that either molecule promotes atherogenesis by altering lipid metabolism because their deletion from EC did not influence cholesterol or triglyceride levels in plasma (Table 1). Collectively, these data indicate that GATA4–TWIST1 signaling promotes EC proliferation, inflammation, and lesion formation at low shear regions of arteries.

**Table 1.**
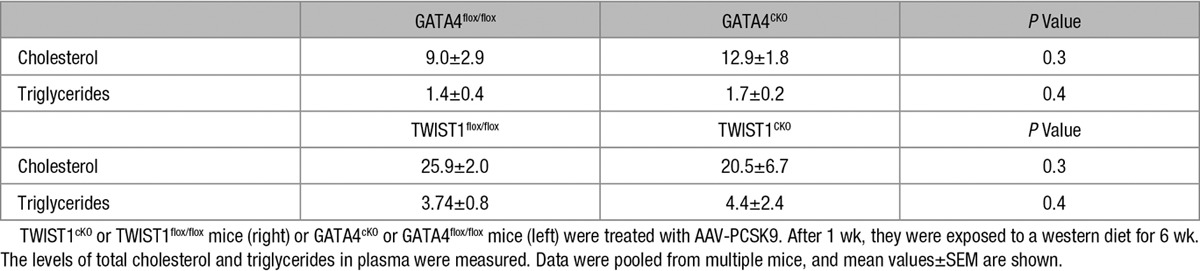
Deletion of TWIST1 or GATA4 From Endothelial Cells Did Not Alter Plasma Lipids

## Discussion

TWIST1 can be regulated by mechanical force during *Drosophila* development^39^ and in response to tumor stiffness.^40^ Here, we demonstrate that mechanical forces also regulate TWIST expression in vascular endothelial cells. Specifically, low shear stress induced TWIST1 in EC via the transcription factor GATA4, thereby enriching TWIST expression at atheroprone sites of arteries. TWIST1 expression contributed to atherosclerosis by enhancing vascular inflammation and driving EC proliferation associated with vascular leakiness. Thus, although TWIST is a central coordinator of embryogenesis, this transcription factor also contributes to the initiation of focal atherosclerosis in adult arteries.

The first part of our study focused on the regulation of TWIST1 by shear stress using in vitro systems and modified murine carotid arteries. Notably, it was observed that TWIST was induced in EC exposed to low-magnitude shear stress with uniform direction but was not enhanced in cells under low oscillatory shear. The mechanism underlying the specificity of TWIST1 induction by low shear stress is uncertain, but it is plausible that unidirectional and oscillatory flow activates different sets of mechanoreceptors that have divergent effects on TWIST1 expression. Our findings are consistent with previous demonstrations that low unidirectional and low oscillatory shear have distinct effects on vascular physiology. Of particular relevance, the imposition of low shear stress in carotid arteries of hypercholesterolemic mice leads to the formation of atherosclerotic plaques with features of instability including a thin fibrous cap and inflammation, whereas the imposition of low oscillatory shear induces stable lesions.^30^ Thus, it will be of interest in future studies to determine whether the induction of TWIST by low shear stress contributes to subsequent plaque inflammation and instability.

A combination of gene silencing, chromatin immunoprecipitation, and genetic deletion studies demonstrated that GATA4 is required for the induction of TWIST by low shear. However, although GATA4 and TWIST were functionally linked in EC exposed to low shear, they were uncoupled in cells exposed to low oscillatory shear where GATA4 was induced in the absence of TWIST expression. This observation indicates that GATA4 is necessary but not sufficient for the induction of TWIST1, suggesting the involvement of other factors that are activated under specific shear stress environments. Notch is a candidate coregulator of TWIST1 because it promotes TWIST expression during development^35,36^ and was induced specifically by low shear stress in murine carotid arteries. This model explains the expression of TWIST1 under low shear (where GATA4 and Notch are both regulated) and its absence under low oscillatory shear (where GATA4 was activated without Notch). Future studies are required to understand the mechanical regulation of GATA4/Notch cross talk and whether these factors cooperate to induce TWIST1 at atheroprone regions. A further outstanding question relates to the mechanoreceptors that regulate TWIST induction in response to low shear stress. Bone morphogenic proteins are candidates because they can be upregulated by low shear stress^41^ and cross talk with TWIST1.^42^ Their potential interaction with TWIST1 in vascular EC should now be investigated.

The second part of our study focused on the function of TWIST1 in EC. Using conditional knockout approaches, it was demonstrated that deletion of GATA4 or TWIST1 from EC significantly reduced lesion formation at low shear stress sites in hypercholesterolemic mice. GATA4–TWIST1 signaling in EC drives atherosclerosis through several mechanisms. First, GATA4 and TWIST1 activation was shown to enhance focal endothelial inflammatory activation, which drives lesion formation by promoting leukocyte recruitment to the vascular wall.^3–7^ Second, GATA4–TWIST1 signaling at low shear stress regions induced EC proliferation, which can enhance the permeability of arteries to cholesterol-containing lipoproteins.^12^ Consistent with this, we observed that GATA4 and TWIST1 enhanced permeability in EC monolayers exposed to low shear stress. The mechanism linking GATA4–TWIST1 signaling to EC proliferation involves cyclins, which were induced by low WSS via a GATA4- and TWIST1-dependent mechanism. EC proliferation at atheroprone sites is regulated by several other signaling intermediaries including p53^9,32^, JNK1,^43^ and miR-126-5p,^11^ and it will be important in future studies to assess how these pathways interact with the GATA4–TWIST1 signaling pathway.

Our study also revealed that GATA4 and TWIST1 induced molecules involved in EndoMT (including Snail, α-smooth muscle actin, and N-cadherin) in EC exposed to low shear. EndoMT is an example of cellular plasticity characterized by a program of morphological and physiological changes that involve loss of apical/basal polarity, disruption of intercellular junctions, increased proliferation, and migration of cells into surrounding tissue.^26^ Thus, activation of EndoMT by GATA4–TWIST signaling may contribute to enhanced proliferation at low shear sites. The canonical function of EndoMT is in the development of valves from EC in the atrioventricular canal^21^; however, EndoMT has also been implicated in disease processes including cerebral cavernous malformations.^44^ Of particular relevance, 2 recent studies demonstrated that EndoMT can be induced by disturbed flow and is a driver of atherosclerosis.^45,46^ Thus, our observation that low shear induces GATA4–TWIST1 signaling provides a molecular mechanism to explain the induction of EndoMT by low shear stress during atherosclerosis. Our study emphasizes the complex heterogenous phenotypes of EC at disease-prone sites. We previously reported that atheroprone sites contain ≈1% senescent cells, which cannot participate in vascular repair because they are irreversibly growth arrested and exhibit defective migration.^32^ Our current study indicates that atheroprone sites also contain TWIST1-positive EC that exhibit enhanced proliferation. Further studies are required to determine whether specific mechanical signatures are required for the induction of EC proliferation or senescence and the interplay between these 2 populations during atherogenesis.

Our study revealed that the function of TWIST is, at least in part, conserved in developing vasculature and atheroprone regions of adult arteries because it enhances EC proliferation in both contexts. However, the consequence of TWIST activation differs between embryogenic and adult arteries because EC proliferation has an essential role in vascular development but promotes lesion initiation in mature arteries by enhancing permeability to lipoproteins. Thus, the induction of TWIST by low shear stress may be an example of antagonistic pleiotropy because it has beneficial effects during development but contributes to arterial disease in adults. Although EC in developing vasculature are phenotypically distinct from those in adult vessels, our study reveals that some aspects of EC physiology, including TWIST induction of proliferation, are conserved between embryonic and adult tissues. This is consistent with the demonstration that several other genes (eg, bone morphogenic proteins^41^ and Notch^47^) and processes (eg, EndoMT^45,46^) with a well-recognized role in development also contribute to focal EC dysfunction and atherosclerosis. We suggest therefore that the behavior of EC at atheroprone sites is a reflection of an early developmental stage and that developmental genes including GATA4 and TWIST1 could be novel therapeutic targets in atherosclerosis.

## Sources of Funding

This study was funded by the British Heart Foundation (RG/13/1/30042).

## Disclosures

None.

## Supplementary Material

## Novelty and Significance

### What Is Known?

- Atherosclerosis develops at branches and bends of arteries exposed to disturbed patterns of blood flow.
- Endothelial cells at atheroprone sites display enhanced proliferation, permeability, inflammation, and expression of mesenchymal genes.
