# Supplementary material for: TWIST1 Integrates Endothelial Responses to Flow in Vascular Dysfunction and Atherosclerosis
Source: Circ Res. 2016 Jul 21;119(3):450–62. doi: 10.1161/CIRCRESAHA.116.308870 (PMC4959828; doi:10.1161/CIRCRESAHA.116.308870)
Supplement: Supplementary file 3 [file res-119-450-s003.pdf]

EXPANDED METHODS

Antibodies and reagents. Antibodies targeting human and murine TWIST1 (ABD29, Millipore), GATA4 (PA1-102, Thermoscientific) and, Snail (ab180714, Abcam), PCNA (ab 18197, Abcam), , CD31 (102514, Biolegend), Ki67 (ab15580, Abcam), Activated-Notch 1 (ab8925) were obtained commercially. AlexaFluor-conjugated secondary antibodies, TO-PRO-3 and aqueous mounting media (ProLong® Gold Antifade Mountant) were from Invitrogen. All other reagents were from Sigma-Aldrich unless otherwise stated.

Mouse lines. Mice were housed under specific-pathogen free conditions and studied according to UK Home Office Regulations and the UK Animals (Scientific Procedures) Act 1986. Mice were weaned at 4 weeks of age and maintained on a normal chow diet. Mice between 2 and 3 months of age were used for experimentation in groups of at least 4 animals. Littermates were used as controls. TWIST1 and GATA4 mice were not back crossed onto a C57BL/6 background for 6 generations. For cell tracing studies, transgenic Rosa26-tdTomato[1] mice were crossed with endothelial-SCL-Cre-ER^T mice containing a tamoxifen-inducible EC-specific Cre[2]. Mice with Tie2-specific deletion of TWIST1 ($TWIST1^{cKO}$) were generated by crossing Tie2-Cre expressing mice (Jackson Laboratory stock #004128) with TWIST1 floxed mice ($TWIST1^{flox|flox}$)[3]. Mice with EC deletion of GATA4 ($GATA4^{cKO}$) were generated by crossing endothelial-SCL-Cre-ER^T mice with GATA4 floxed mice ($GATA4^{flox|flox}$). To activate Cre, tamoxifen was administered for 5 consecutive days (160 mg/kg). Constrictive cuffs with an internal diameter of 400 µm tapering to 200 µm over 1.5 mm were manufactured from PTFE and applied to the right carotid artery of isofluorane-anaesthetized mice following published methods[4, 5].

En face staining of murine endothelium. The expression levels of specific proteins were assessed in EC at regions of the inner curvature (susceptible site) and outer curvature (protected site) of murine aortae and in carotid arteries by *en face* staining. Animals were killed by I.P injection of pentobarbital or by isofluorane overdose. Aortae were perfused *in situ* with PBS (at a pressure of approximately 100 mm Hg) and then perfusion-fixed with 4% Paraformaldehyde prior to harvesting. Fixed aortae were tested by immunostaining using specific primary antibodies and Alexafluor568-conjugated secondary antibodies (red). EC were identified by co-staining using anti-CD31 antibodies conjugated to the fluorophore FITC (green). Nuclei were identified using a DNA-binding probe with far-red emission (To-Pro-3). Stained vessels were mounted prior to visualization of endothelial surfaces *en face* using confocal laser-scanning microscopy (Zeiss LSM510 NLO inverted microscope). Isotype-matched monoclonal antibodies raised against irrelevant antigens or pre-immune rabbit sera were used as experimental controls for specific staining. The expression of particular proteins at each site was assessed by quantification of fluorescence intensity for multiple cells (at least 50 per site) using Image J (1.49p) and calculation of mean fluorescence intensities with standard error of the mean.

Lesion analysis.

Hypercholesterolemia was induced in mice by a single Intraperitoneal injection of adenoassociated virus containing PCSK9 cDNA (rAAV8-D377Y-mPCSK9; 4.9 $\times 10^{11}$ pfu) and subsequent exposure to a high fat Western diet (Western RD (P) Diet,829100; SDS) for 6 weeks[6]. The diet consisted of (w/w) Milk Fat Anhydrous (20%), cholesterol (0.15%), sucrose (33.94%), cornstarch (5%), corn oil (1%), cellulose (5%), casein (19.5%), choline chloride (0.2%), L-Cystine (0.3%), Calcium Carbonate (0.4%), Choline Bitartrate (0.2%), Antioxidant (0.01%), AIN-76A-MX (3.5%), AIN-76A-VX (1%) total fat content (21.4%). Analysis for lipoprotein profiles

and serum total cholesterol and triglycerides, and *en face* lipid staining was carried out using Oil Red O as described[7]. After termination, perfusion fixation was performed using 10% Formalin at a pressure of 110 cm water for 5 min via a cannula inserted into the left ventricle. Each aorta was isolated and adventicial fat was removed before staining using Oil Red O. After cutting along the outer curvature, the aorta was mounted and imaged. Lesion coverage in aortae was analyzed as previously described (Steiner et al., 2014). Lesion coverage was calculated as a percentage of total aortic area.

Isolation of EC from porcine aortae. Pig aortas from 4-6 month old animals (weight approximately 80kg) were obtained immediately after slaughter from a local abattoir. They were cut longitudinally along the outer curvature to expose the lumen. EC exposed to high (outer curvature) or low (inner curvature) WSS were harvested using collagenase (1 mg/ml for 10 minutes at room temperature) prior to gentle scraping. RNA was extracted using an RNeasy MiniKit (Qiagen) and concentrated using an RNeasy MinElute Cleanup kit (Qiagen) and the purity and integrity of total RNA samples was assessed using a Bioanalyser (Agilent).

EC culture and exposure to WSS. HUVEC and PAEC were isolated using collagenase digestion. EC were cultured on 1% gelatin and maintained in M199 growth medium (HUVEC) or DMEM (PAEC) supplemented with foetal bovine serum (20%), L-glutamine (4 mmol/L), endothelial cell growth supplement (30 µg/ml), penicillin (100 U/ml), streptomycin (100 µg/ml) and heparin (10 IU/ml). EC at passage 3-5 were cultured until confluent in 6 well plates and exposed to flow using an orbital shaking platform (PSU-10i; Grant Instruments) housed inside a cell culture incubator. The radius of orbit of the orbital shaker was 10mm and the rotation rate was set to 210 rpm. This motion caused swirling of the culture medium over the cell surface generating low tangential WSS at the centre and high uniform WSS at the periphery. Alternatively, HUVEC were cultured on Ibidi® gelatin-coated µ-Slides (Ibidi GmbH) until they reached confluency. Flowing medium was then applied using the Ibidi® pump system to generate low (4 dyn/cm^2), low oscillatory (+/- 4 dyn/cm^2, 0.5 Hz) or high (13 dyn/cm^2) WSS. The slides and pump apparatus were enclosed in a cell culture incubator warmed to 37°C.

RNA interference. Cell cultures were transfected with siRNA sequences that are known to silence TWIST1 (Silencer® Select S14523, Ambion® and L-006434-00-0005 ON-TARGETplus SMARTpool, Dharmacon) or GATA4 (Silencer® Select s5603, Ambion® and L-008244-00-0005 ON-TARGETplus SMARTpool, Dharmacon) using the Lipofectamine® RNAiMAX transfection system (13778-150, Invitrogen) following the manufacturer's instructions. Two different siRNAs were used to test each gene to ensure specificity. Final siRNA concentration used was 25nM[8]. After knockdown, cells were then incubated in complete M199 growth medium for 2 h before exposure to flow. Non-targeting scrambled sequences were used as a control (D-001810-01-50 ON-TARGETplus Non targeting siRNA#1, Dharmacon).

Comparative real time PCR. RNA was extracted using the RNeasy Mini Kit (74104, Qiagen) and reverse transcribed into cDNA using the iScript cDNA synthesis kit (1708891, Bio-Rad). The levels of human, porcine or zebrafish transcripts were assessed using quantitative real time PCR (qRT-PCR) using gene-specific primers (Supplementary Table 1). Reactions were prepared using SsoAdvanced universal SYBR®Green supermix (172-5271, Bio-rad) and following the manufacturer's instructions, and were performed in triplicate. Relative gene expression was calculated by comparing the number of thermal cycles that were necessary to generate threshold amounts of product. Fold changes were calculated using the

ΔΔCt method. Data were pooled from at least three independent experiments and mean values were calculated with SEM.

Western blotting Total cell lysates were isolated using lysis buffer (containing 2% SDS, 10% Glycerol and 5% β-mercaptoethanol). Western blotting was carried out using specific antibodies against GATA4 (Sc 1237 (C-20), Santa Cruz), TWIST1 (ABD29, Millipore) and Calnexin (610524, BD Transduction Laboratories) horse radish peroxidase-conjugated secondary antibodies obtained commercially from Dako and chemiluminescent detection was carried out using ECL Prime® (GE Healthcare). Membranes were imaged using the C-DiGit scanner (LI-COR Biosciences).

Immunofluorescent staining of cultured EC. The expression levels of proteins were assessed by immunostaining using specific antibodies followed by widefield fluorescence microscopy (LeicaDMI4000B). HUVEC were fixed with Paraformaldehyde (4%) and permeabilised with Triton X-100 (0.1%). Following blocking with goat serum for 30 min monolayers were incubated for 16 h with primary antibodies against TWIST1, GATA4, Snail, or PCNA and AlexaFluor488- or Alexafluor568-conjugated secondary antibodies. Nuclei were identified using the DNA-binding probe DAPI (Sigma). Image analysis was performed using Image J software (1.49p) to calculate average fluorescence. Isotype controls or omission of the primary antibody was used to control for non-specific staining.

Promoter analysis and chromatin immunoprecipitation (ChIP). Genomic sequences (www.epd.vital-it.ch) were interrogated for putative GATA4 binding sites (A/T) GATAA(G) using TFSEARCH data base (http://www.cbrc.jp/research/db/TFSEARCH.html). For ChIP, HUVECs were fixed using formaldehyde (1% for 10 min). ChIP was carried out using the SimpleChIP®Plus Enzymatic Chromatin IP Kit (#9005; Cell signalling) following the manufacturer's instructions. ChIP-grade GATA4 antibodies (C-20 sc-1237, Santa Cruz) or isotype-matched IgG control were used. Precipitation of specific genomic DNA fragments was assessed by qRT-PCR using primers that amplify regions of the TWIST1 promoter.

Assay of permeability. The permeability of EC monolayers exposed to flow was determined using rhodamine-labelled albumin[9]. HUVEC were cultured in transwell inserts overnight and then exposed to orbital shaking for 72 h. The media in the upper compartment was then replaced with 10% serum-supplemented DMEM containing 1% BPA and rhodamine-labeled albumin (1 mg/ml). Media in the lower compartment was sampled at 1 h and fluorescence was measured using a fluorimeter (Varioskan, Thermoscientific) with excitation at 570 nm and emission at 600 nm. Data were pooled from five experiments and mean concentrations of rhodamine albumin +/- SEM were calculated.

Zebrafish lines. The experiments were performed in accordance with the UK Animal (Scientific Procedures) Act, 1986 and the NIH Guide for the Care and Use of Laboratory Animals. Adult zebrafish were maintained on a 14 h light / 10 h dark cycle at 28°C in UK Home Office approved facilities in the Bateson Centre at the University of Sheffield. Studies were carried out on wild-type zebrafish or transgenic zebrafish lines, *Tg(fli1:EGFP)* (endothelial EGFP), *Tg(kdrl:NLS-EGFP)* (endothelial nuclear EGFP) or *Tg(gata1:dsRed)* (red blood cell expression of dsRed). To generate mutants, a Cas9 expression plasmid (Addgene) was linearised with ApaI and mRNA for injection was generated with mMESSAGE mMACHINE SP6 Kit (Life Technologies). For gRNA synthesis, a *twist1b* CRISPR target site was selected with

the sequence 5'-GG-TTCCGATAGCTCGACCCT-NGG-3' which contains a BsaJI restriction enzyme site used for genotyping. An ultamer of the sequence (AAAGCACCGACTCGGTGCCACTTTTTCAAGTTGATAACGGACTAGCCTTATTTT AACTTGCTATTTCTAGCTCTAAAACAGGGTCGAGCTATCGGAATCCTATAGTGAG TCGTATTACGC) for gRNA was *in vitro* transcribed with the MEGAshortscript T7 kit (Life technologies). This was injected at a concentration of 2.4 μg/μl along with 0.5 μg/μl Cas9 mRNA into newly fertilized embryos. Injected embryos were raised to maturity (F0) and their progenies were genotyped for *twist1b* mutation. The embryos harbouring mutation were raised into adults (F1) and genotyped by PCR analysis of DNA from fin clips.

Gain- and loss-of-function studies in zebrafish. *Twist1a* translation blocking morpholino (5'-ACCTCTGGAAAAGCTCAGATTGCGG-3') was purchased from Gene Tools LLC (Philomath, OR, USA) and injected at a concentration of 0.5mM and in a volume of 1nl into one cell stage embryos. To generate *twist1b* mRNA, the *twist1b* coding sequence was cloned into pCS2+ expression vector. The plasmid was linearized with NotI and transcribed with SP6 polymerase. The SP6 mMessage Machine kit (Ambion, Texas, USA) was used for *in vitro* transcription. Transgenic or mutant embryos were injected at the 1-cell stage with MO or capped messenger RNA using a Narishige IM-300 micromanipulator. Live embryos were analysed embedded in 0.7% low melting agarose using spinning disk confocal microscope (Perkin Elmer) and Leica stereoscope (M165FC)

In situ hybridisation and immunofluorescence staining of zebrafish embryos*.* The whole mount *in situ* hybridization of zebrafish embryos was performed using digoxigenin (DIG) labelled *twist1a*, *twist1b*, *twist2* probes synthesized using a DIG labelling mix (Roche) and T7 or SP6 polymerase (Roche). Imaging was performed using an Olympus FV1000 laser scanning confocal microscope.

Statistics. Differences between samples were analysed using an unpaired or paired Student's t-test or ANOVA (*p<0.05, **p<0.01, ***p<0.001).

REFERENCES FOR EXPANDED METHODS

1. Madisen L, Zwingman TA, Sunkin SM, Oh SW, Zariwala HA, Gu H, Ng LL, Palmiter RD, Hawrylycz MJ, Jones AR, Lein ES and Zeng H. A robust and high-throughput Cre reporting and characterization system for the whole mouse brain. *Nature Neurosci*. 2010;13:133-140.
2. Gothert JR, Gustin SE, van Eekelen JA, Schmidt U, Hall MA, Jane SM, Green AR, Gottgens B, Izon DJ and Begley CG. Genetically tagging endothelial cells in vivo: bone marrow-derived cells do not contribute to tumor endothelium. *Blood*. 2004;104:1769-1777.
3. Li J, Liu CH, Sun Y, Gong Y, Fu Z, Evans LP, Tian KT, Juan AM, Hurst CG, Mammoto A and Chen J. Endothelial TWIST1 promotes pathological ocular angiogenesis. *Investig Ophthalmol Vis Sci*. 2014;55:8267-8277.
4. Cheng C, Tempel D, van Haperen R, van der Baan A, Grosveld F, Daemen MJ, Krams R and de Crom R. Atherosclerotic lesion size and vulnerability are determined by patterns of fluid shear stress. *Circulation*. 2006;113:2744-2753.
5. Cuhlmann S, Van der Heiden K, Saliba D, Tremoleda JL, Khalil M, Zakkar M, Chaudhury H, Le AL, Mason JC, Udalova I, Gsell W, Jones H, Haskard DO, Krams R and Evans PC. Disturbed Blood Flow Induces RelA Expression via c-Jun N-Terminal Kinase 1 A Novel Mode of NF-kappa B Regulation That Promotes Arterial Inflammation. *Circ Res*. 2011;108:950-959.
6. Bjorklund MM, Hollensen AK, Hagensen MK, Dagnaes-Hansen F, Christoffersen C, Mikkelsen JG and Bentzon JF. Induction of Atherosclerosis in Mice and Hamsters Without Germline Genetic Engineering. *Circ Res*. 2014;114:1684-1689.
7. Steiner T, Francescut L, Byrne S, Hughes T, Jayanthi A, Guschina I, Harwood J, Cianflone K, Stover C and Francis S. Protective Role for Properdin in Progression of Experimental Murine Atherosclerosis. *Plos One*. 2014;9.
8. Zografou S, Basagiannis D, Papafotika A, Shirakawa R, Horiuchi H, Auerbach D, Fukuda M and Christoforidis S. A complete Rab screening reveals novel insights in Weibel-Palade body exocytosis. *J Cell Sci*. 2012;125:4780-4790.
9. Warboys CM, Berson R, Mann GE, Pearson JD and Weinberg PD. Acute and chronic exposure to shear stress have opposite effects on endothelial permeability to macromolecules. *Am J Physiol-Heart Circ Physiol*. 2010;298:H1850-H1856.

Supplementary Table PCR primer sequences

Gene	Forward primer	Reverse primer
GATA4 (human)	TCCCAGACGTTCTCAGTCAG	GGAGCTGGTCTGTGGAGACT
TWIST1 (human)	CGGACAAGCTGAGCAAGAT	CTGGAGGACCTGGTAGAGGA
TWIST1 promoter (human)	AGCAATCCCAAATCGGCCC	TGGCAACAGCTTCTACACAGT
SLUG (human)	CTGGCCAAACACAAGCAG	ACCCAGGCTCACATATTCCT
N-cadherin (human)	GCACAGATGTGGACAGGATT	CAGCACAAGGATAAGCAGGA
α-SMA (human)	TTTCAGCTTCCCTGAACACCA	GGGCAACACGAAGCTCATTG
Snail (human)	TGCAGGACTCTAATCCAGAGTTT	GACAGAGTCCCAGATGAGCA
Cyclin D1 (human)	TGA CCC CGC ACG ATT TCA TT	TGA GGC GGT AGT AGG ACA GG
Cyclin G2 (human)	TTG GAC AGG TTC TTG GCT CT	TCA ACT ATT CTA GCA GCC AGC
Cdk4 (human)	CAA TGT TGT CCG GCT GAT GG	GCT CAA ACA CCA GGG TTA CC
VCAM-1 (human)	CATTGACTTGCAGCACCACA	AGATGTGGTCCCCTCATTCG
ICAM-1 (human)	CACAAGCCACGCCTCCCTGAACCTA	TGTGGGCCTTTGTGTTTTGATGCTA
CD31 (human)	GGTGTTGGTGGAAGGAGTG	GGGACAGAACAGTTGACCCT
Piezo1 (human)	ATCGCCATCATCTGGTTCCC	TGGTGAACAGCGGCTCATAG
VEGFR2 (human)	CTCGGGTCCATTTCAAATCT	GCTGTCCCAGGAAATTCTGT
HPRT (human)	TTGGTCAGGCAGTATAATCC	GGGCATATCCTACAACAAC
GATA4 (porcine)	GGAAGGCAGAGAGTGTGTCA	GCTGATGCCATTCATCTTGT
TWIST1 (porcine)	GGGAGTCCGCAGTCCTAC	TGGATCTTGCTCAGCTTGTC
twist1a (zebrafish)	CGCGTTTTCTGTGTGGAGAA	CCGAGAATCATGCTGCATCA
twist1b (zebrafish)	CGCTTGGTCCATGTCTGC	CTCGTGTTTTCCCAGCTCAC
twist2 (zebrafish)	CGTCCTGATAATGCCGAACG	GCCACGTCACTGAGGTTTAG

SUPPLEMENTAL MATERIAL

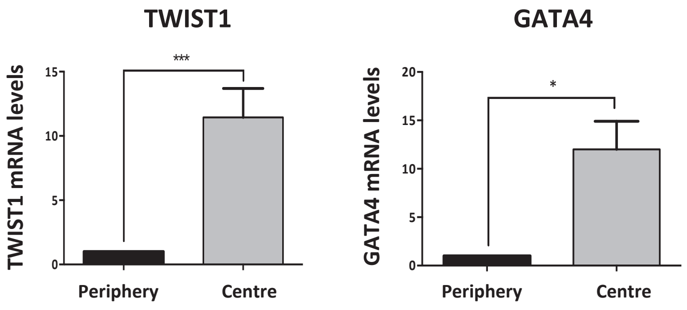

Online Figure I Low shear induced TWIST1 and GATA4 in cultured PAEC.
PAEC were exposed to orbital flow to generate low (Centre) or high (Periphery) wall shear stress (WSS). After 72 h, transcript levels were quantified by qRT-PCR and mean values +/- SEM are shown.

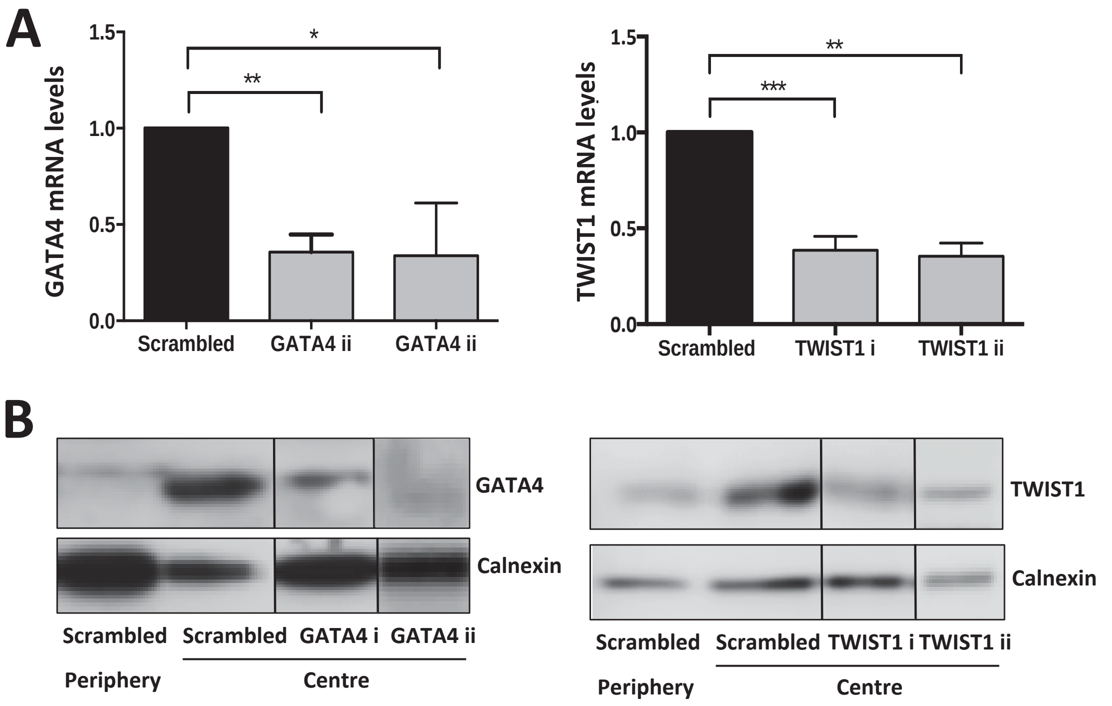

Online Figure II Validation of TWIST1 and GATA4 gene silencing.
Silencing of TWIST1 or GATA4 was performed using two different siRNAs for each gene (designated i and ii). HUVEC were treated with siRNA targeting GATA4 or TWIST1, or with scrambled non-targeting siRNA as a control. After knockdown, cells were then cultured in 6 well plates prior to the application of orbital flow for 72 h. (A) Transcript levels of TWIST1 (left) or GATA4 (right) in cells at the centre of the well (low WSS) were quantified by qRT-PCR. Data were pooled from 3 independent experiments and mean levels +/- SEM are shown. (B) Expression of TWIST1 and GATA4 protein at the centre of the well (low WSS) was determined by Western blotting using anti-Calnexin antibodies to control for total protein levels.

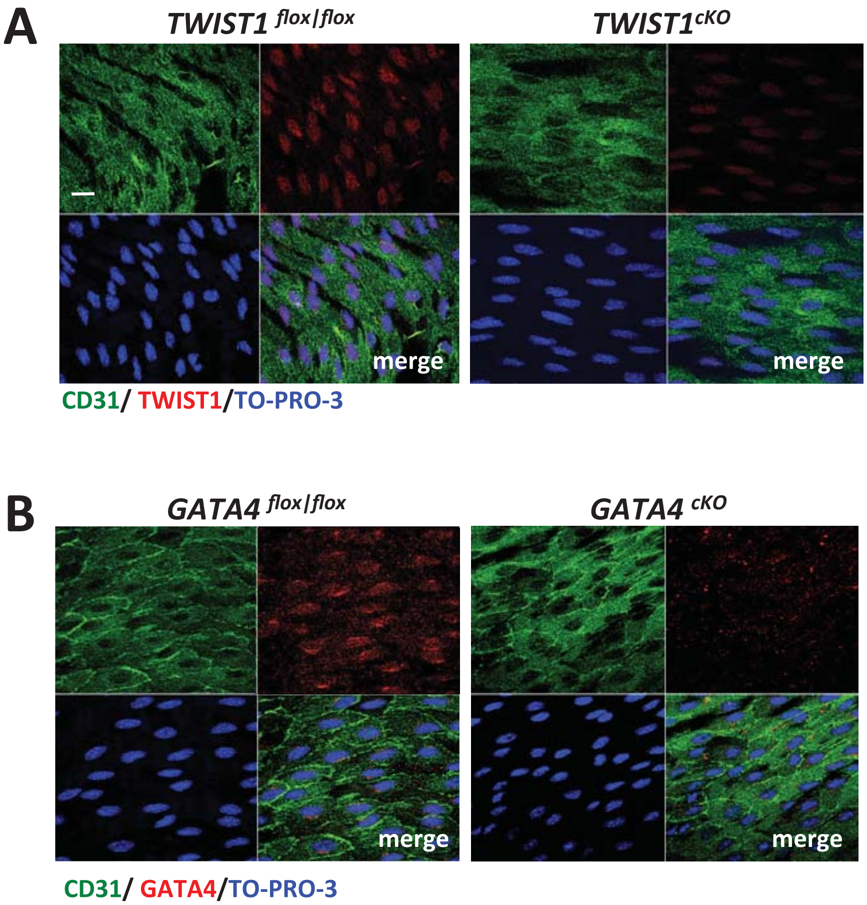

Online Figure III Validation of genetic targeting of TWIST1 and GATA4 in endothelial cells of transgenic mice.
(A) TWIST1 floxed mice (TWIST1$^{flox/flox}$) were crossed with Tie2-Cre transgenics (generating TWIST1^{cKO}). (B) GATA4$^{flox/flox}$ mice were crossed with endothelial-SCL-Cre-ER^{T} transgenics followed by 5 days of tamoxifen treatment (generating GATA4^{cKO}). (A, B) To validate genetic deletion of TWIST1 and GATA4 from EC in the descending aorta *en face* staining was performed using anti-TWIST1 or anti-GATA4 antibodies (red). Co-staining was carried out using anti-CD31 antibodies (green) and the nuclear counter stain TOPRO3 (blue). Representative images are shown. Scale bar 10 µm.

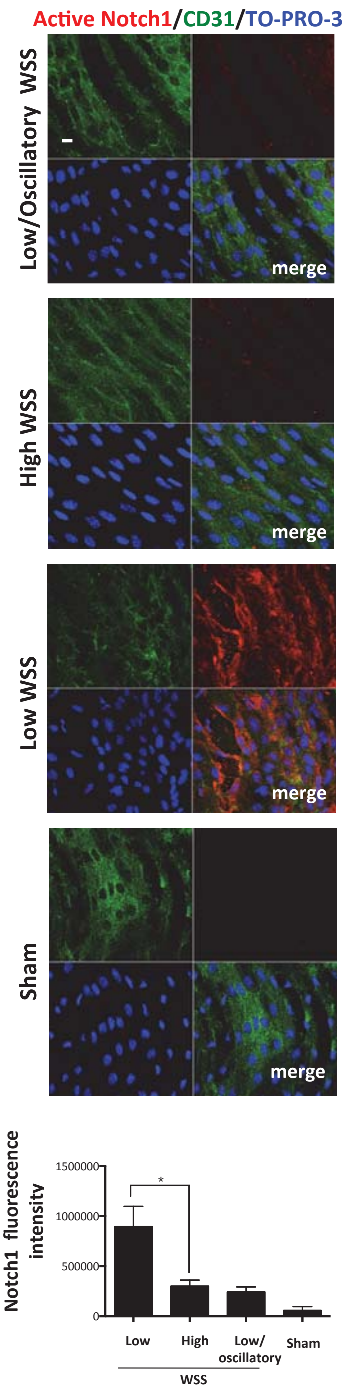

Online Figure IV Low shear stress induced Notch1 in experimental carotid arteries.

Flow-altering, constrictive cuffs were placed on the right carotid arteries of C57BL/6 mice. They generated anatomically distinct regions exposed to low, high and low oscillatory wall shear stress (WSS; as indicated). Right (experimental) and left (sham-operated) carotid arteries were harvested after 14 days and *en face* staining was performed using anti-Notch1 antibodies (red), anti-CD31 antibodies conjugated to FITC (green) and the nuclear counter stain TO-PRO-3 (blue). Representative images and quantitation of Notch1 expression (mean +/- SEM) are shown. Scale bar, 10 µm. Data were pooled from five-six independent experiments. *$p<0.05$ using a one-way ANOVA.

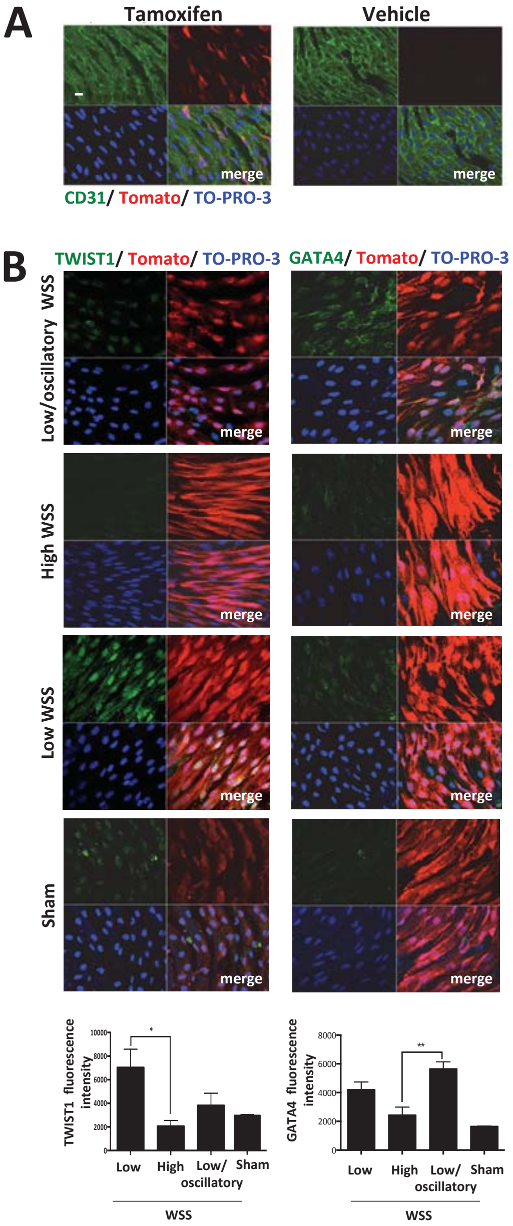

Online Figure V **Low shear stress induced TWIST1 in fully differentiated EC *in vivo.*** Transgenic SCL-Cre-ER^{T}/R26RtdTomato mice were used to track endothelial cells. The administration of tamoxifen induces nuclear localization of Cre, which subsequently excises a floxed STOP signal from the tdTomato promoter thus inducing tdTomato expression. (A) To validate the system, SCL-Cre-ER^{T}/R26RtdTomato mice were treated with tamoxifen for 5 consecutive days or with vehicle as a control. After a further 7 days, carotid arteries were harvested and *en face* staining was performed using anti-CD31 antibodies (green), and the nuclear counter stain TOPRO3 (blue). TdTomato was expressed in the majority of EC in mice treated with tamoxifen but not in vehicle-treated mice. (B) SCL-Cre-ER^{T}/R26RtdTomato mice were treated with tamoxifen for 5 consecutive days. One week later, flow-altering constrictive cuffs were placed on the right carotid arteries to generate regions exposed to low, high and low oscillatory wall shear stress (WSS; as indicated). Right (experimental) and left (sham-operated) carotid arteries were harvested after 14 days and *en face* staining was performed using anti-TWIST1 or anti-GATA4 antibodies (green), and the nuclear counter stain TO-PRO-3 (blue). Representative images and quantitation of TWIST1 or GATA4 expression (mean +/- SEM) are shown. Scale bars, 10 µm. *p<0.05 using a one-way ANOVA.

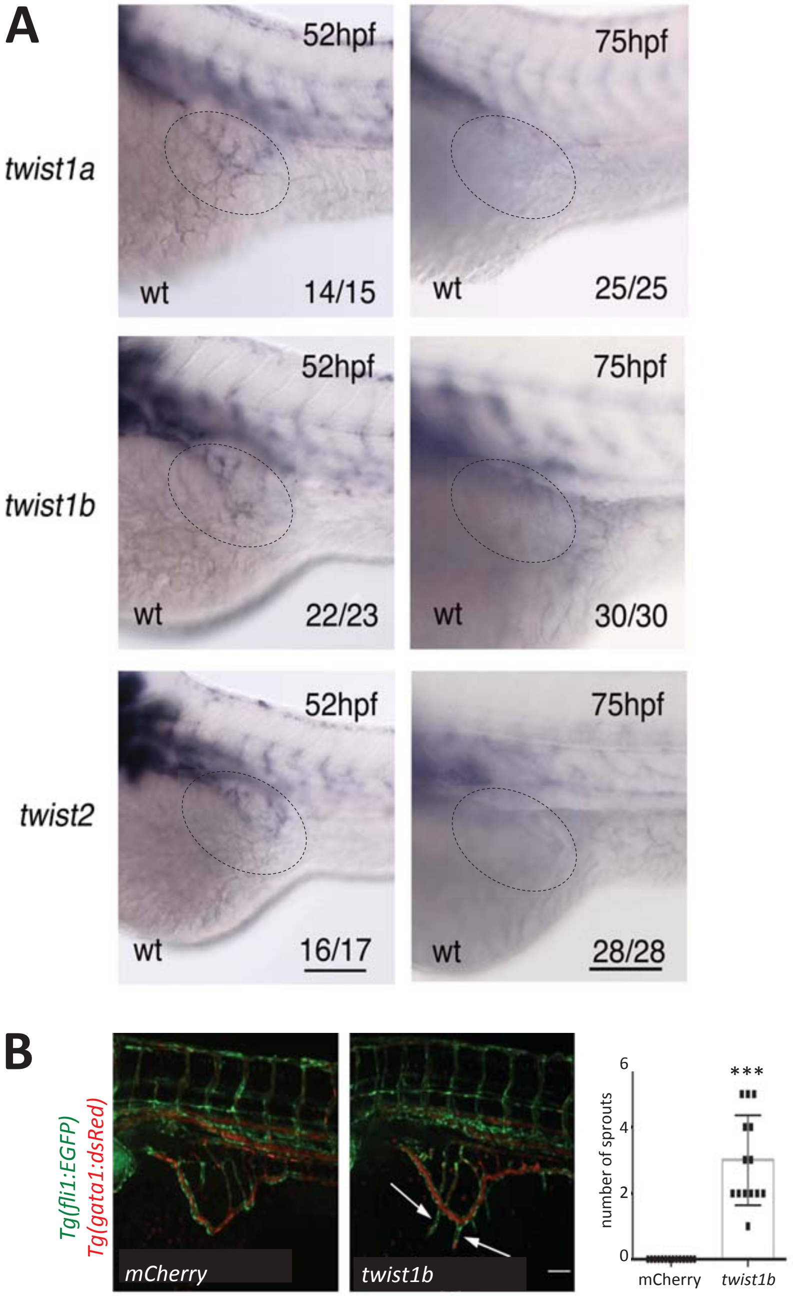

Online Figure VI ***twist1* regulated sub-intestinal vein sprouting in embryos.** (A) *In situ* hybridisation analysis of *twist1a, twist1b* or *twist2* expression in embryos at 52 or 75 hpf. Scale bar, 100 μm. SIV region is indicated using a broken line. Data shown are representative of the majority of embryos analysed (proportion indicated lower right in each panel). (B) Embryos (*Tg(fli1:EGFP;gata1:dsRed)*) were treated with *twist1b* mRNA (to enforce expression) or with mCherry mRNA as a control. They were studied at 75 hpf (flow was established as evidenced by *gata1*-positive red blood cells). Confocal microscopy was used to visualise angiogenic sprouts (arrows). The number of angiogenic sprouts was quantified for multiple embryos and mean values +/- SEM are shown. Representative images are shown. Scale bar, 500 μm. *** p<0.001 using an unpaired t-test.

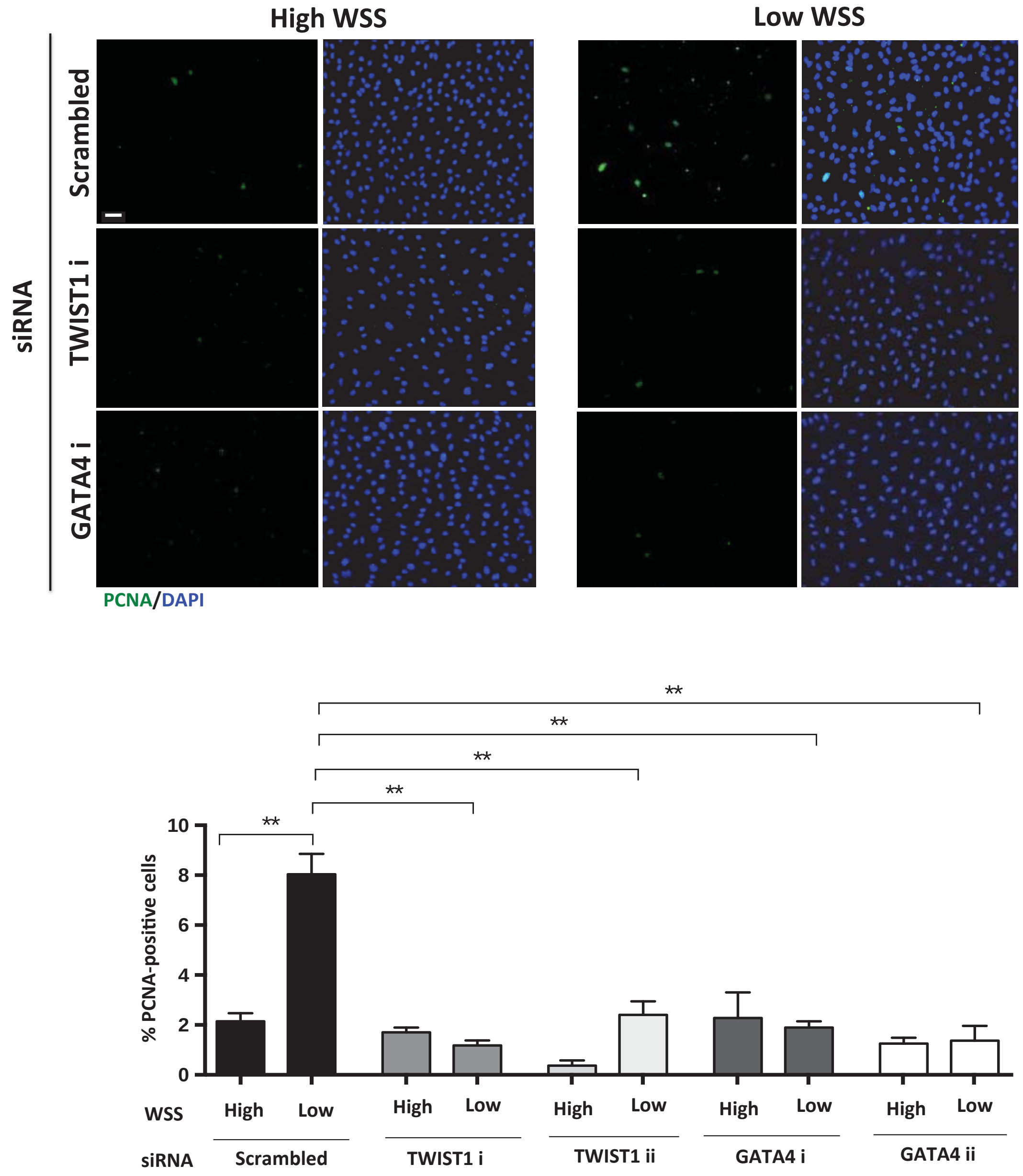

Online Figure VII TWIST1 and GATA4 promoted proliferation in EC exposed to low shear stress using a parallel plate system.
HUVEC were treated with two different siRNAs targeting TWIST1 or GATA4 (designated i and ii), or with scrambled non-targeting siRNA. Transfected cells were exposed to low or high WSS for 72 h using a parallel plate system. Cell proliferation was quantified by immunofluorescent staining using anti-PCNA antibodies and co-staining using DAPI. Images are representative of those generated in three independent experiments using one version of the gene-specific siRNA or scrambled control sequences. The % PCNA-positive cells were calculated for multiple fields of view in at least three independent experiments and mean values +/- SEM are shown. Scale bar, 50 µm.

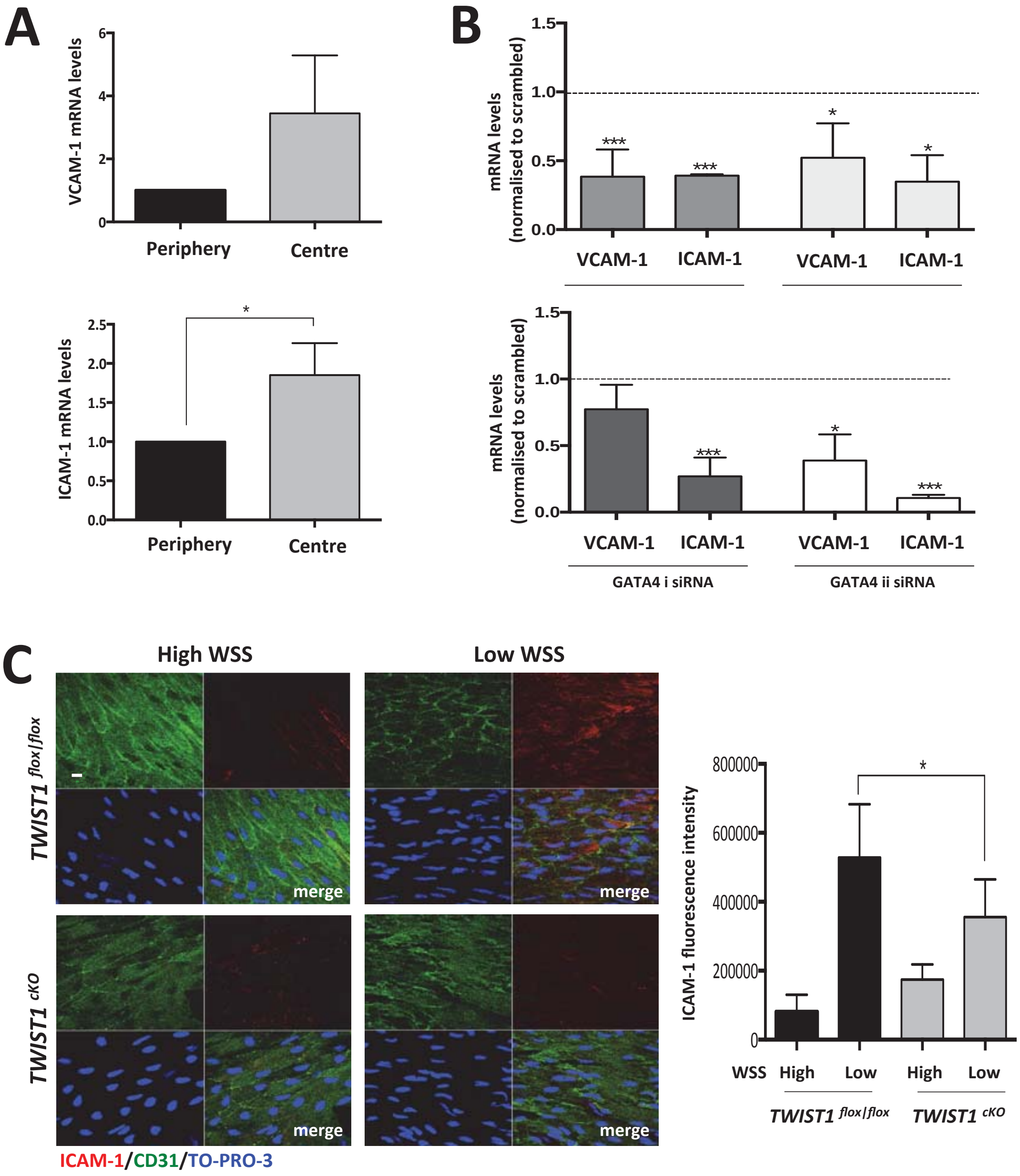

Online Figure VIII TWIST1 and GATA4 induced inflammatory genes in EC exposed to low shear stress.

(A) HUVEC were cultured in 6 well plates prior to exposure to orbital flow to generate low (Centre) or high (Periphery) wall shear stress for 72 h. The expression of VCAM-1 and ICAM-1 was quantified using qRT-PCR. (B) HUVEC were treated with two different siRNAs targeting TWIST1 or GATA4 (designated i and ii), or with scrambled non-targeting siRNA. Transfected cells were exposed to low WSS (centre). The expression of VCAM-1 and ICAM-1 was quantified using qRT-PCR. The expression level in cells transfected with gene-targeting siRNA is presented relative to the expression in cells transfected with scrambled control siRNA (normalised to 1; dotted line). Data were pooled from three independent experiments and mean values +/- SEM are shown. * $p<0.05$, *** $p<0.001$ using an unpaired t-test. (C) The influence of TWIST1 on ICAM-1 expression in EC at low (susceptible) or high (protected) WSS regions of the aorta was studied by *en face* staining using TWIST1^{cKO} or TWIST1$^{flox/flox}$ mice. Staining was performed using anti-ICAM-1 antibodies (red), anti-CD31 antibodies (green) and the nuclear counter stain TO-PRO-3 (blue). Mean fluorescence values were calculated from multiple cells in 5 independent experiments and mean values +/- SEM are shown. Scale bar, 10 μm.

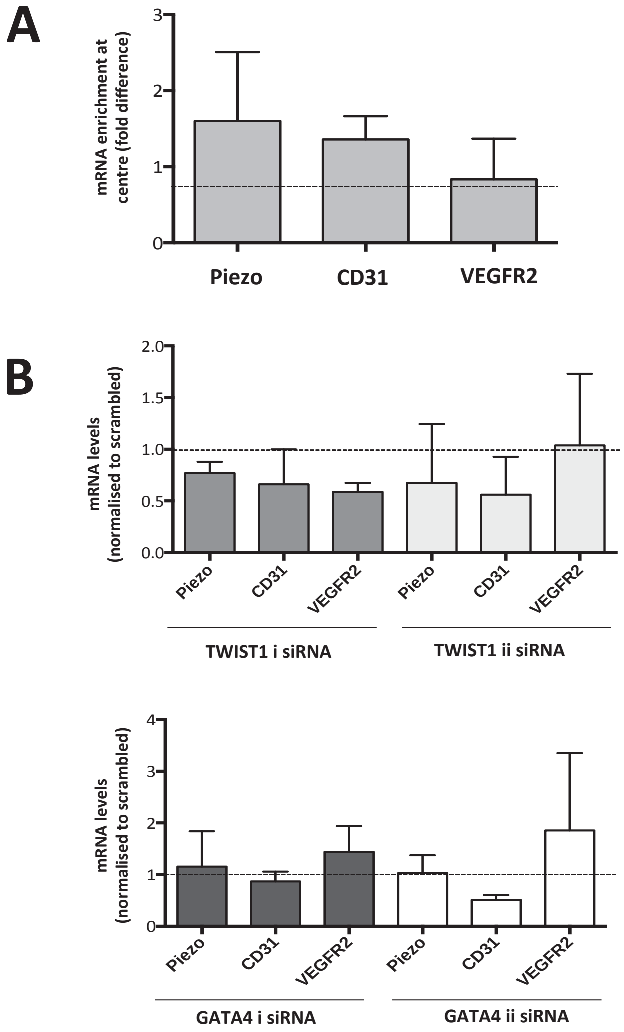

Online Figure IX TWIST1 and GATA4 did not regulate the expression of mechanoreceptors in EC exposed to shear stress.

(A) HUVEC were cultured in 6 well plates prior to exposure to orbital flow to generate low (Centre) or high (Periphery) wall shear stress for 72 h. The expression of Piezo, CD31 and VEGFR2 was quantified using qRT-PCR. (B) HUVEC were treated with two different siRNAs targeting TWIST1 or GATA4 (designated i and ii), or with scrambled non-targeting siRNA. Transfected cells were exposed to low WSS (centre). The expression of Piezo, CD31 and VEGFR2 was quantified using qRT-PCR. The expression level in cells transfected with gene-targeting siRNA is presented relative to the expression in cells transfected with scrambled control siRNA (normalised to 1; dotted line). Data were pooled from three independent experiments and mean values +/- SEM are shown. Data were not significant using an unpaired t-test.

Legends for Online Videos

Online Video I: Intersegmental vessel (ISV) sprouting in control embryos
Online Video II: Enhanced ISV sprouting in twist1b overexpressing embryos

Zebrafish embryos (wild-type, Tg(*fli1-EGFP)* were treated with *twist1b* mRNA or mCherry mRNA (control) and studied at 24-27 hpf, a time where flow is established in trunk vasculature. The migration during ISV sprouting was promoted in embryos overexpressing *twist1b* (Online Video II) in comparison to mCherry mRNA injected control embryos (Online Video I). The movies were taken for the equal duration of 4 hours.
